# Myocardial Bmp2 gain causes ectopic EMT and promotes cardiomyocyte proliferation and immaturity

**DOI:** 10.1038/s41419-018-0442-z

**Published:** 2018-03-14

**Authors:** Belén Prados, Paula Gómez-Apiñániz, Tania Papoutsi, Guillermo Luxán, Stephane Zaffran, José María Pérez-Pomares, José Luis de la Pompa

**Affiliations:** 1 Intercellular Signaling in Cardiovascular Development & Disease Laboratory, Centro Nacional de Investigaciones Cardiovasculares Carlos III (CNIC), Melchor Fernández Almagro 3, 28029 Madrid, Spain; 2 CIBER CV, Madrid, Spain; 3 Aix Marseille Université, Inserm, GMGF UMR_910, 13005 Marseille, France; 4 Department of Tissue Morphogenesis, Max Planck Institute for Molecular Biomedicine, Roentgenstrasse 20, 48149 Muenster, Germany; 5 Department of Animal Biology, Faculty of Science, University of Málaga, Campus de Teatinos s/n, 29071 Málaga, Spain; 6 BIONAND, Centro Andaluz de Nanomedicina y Biotecnología (Junta de Andalucía, Universidad de Málaga), c/ Severo Ochoa No. 25, Campanillas, 29590 Málaga, Spain

## Abstract

During mammalian heart development, restricted myocardial Bmp2 expression is a key patterning signal for atrioventricular canal specification and the epithelial–mesenchyme transition that gives rise to the valves. Using a mouse transgenic line conditionally expressing *Bmp2*, we show that widespread *Bmp2* expression in the myocardium leads to valve and chamber dysmorphogenesis and embryonic death by E15.5. Transgenic embryos show thickened valves, ventricular septal defect, enlarged trabeculae and dilated ventricles, with an endocardium able to undergo EMT both in vivo and in vitro. Gene profiling and marker analysis indicate that cellular proliferation is increased in transgenic embryos, whereas chamber maturation and patterning are impaired. Similarly, forced Bmp2 expression stimulates proliferation and blocks cardiomyocyte differentiation of embryoid bodies. These data show that widespread myocardial Bmp2 expression directs ectopic valve primordium formation and maintains ventricular myocardium and cardiac progenitors in a primitive, proliferative state, identifying the potential of Bmp2 in the expansion of immature cardiomyocytes.

## Introduction

Formation of the primitive cardiac valves begins at E9.5 in mice, when signals from the atrioventricular canal (AVC) myocardium stimulate the adjacent endocardial cells to undergo an epithelial–mesenchyme transition (EMT) and form the valves primordia. This process is patterned and only AVC endocardial cells are competent to respond to these signals and initiate EMT^1^. Studies in mice have revealed the genetic network controlling AVC myocardium patterning, including the T-box transcription factors Tbx2 and Tbx3, which repress chamber-specific gene expression in AVC^2,3^ and Tbx20, which restricts Tbx2 expression to the AVC in a Smad-dependent manner^4^.

Bmp2 (bone morphogenetic protein 2) a transforming growth factor beta (Tgfβ) superfamily member, expressed in AVC myocardium, is sufficient for AVC specification and EMT induction^5–8^. Bmp2 controls AVC myocardial patterning via *Tbx2* activation^9^, and attenuates AVC myocardial proliferation via *n-Myc* repression^10^. Temporal control of BMP2 signalling is crucial for cardiomyocyte differentiation from mouse ES cells (mESC) in vitro. Thus, inhibition of BMP2 signalling before embryoid body (EB) formation, or in mesoderm-committed (Brachyury-T positive) EBs, induces cardiomyogenesis^11^.

We asked whether Bmp2 is able to specify a prospective ventricle as AVC, and what is the effect of Bmp2 on chamber cardiomyocytes. We have generated a transgenic line conditionally expressing Bmp2 and examined the consequences of ectopic Bmp2 expression in heart development. *Nkx2*.*5*^*Cre*^-driven *Bmp2* myocardial overexpression leads to embryonic death at E15.5, and rescues the AVC specification defect of *Bmp2*-null embryos. E14.5 *Nkx2*.*5*^*Cre/+*^*;Bmp2*^*tg/+*^ embryos show enlarged valves and trabeculae, dilated ventricles and ventricular septal defect. Remarkably, transgenic ventricular endocardium is EMT-competent both in vivo and in vitro. Gene profile and marker analysis of *Nkx2*.*5*^*Cre/+*^*;Bmp2*^*tg/+*^ hearts indicated that cardiac cellular proliferation is increased, and while chamber myocardium gene expression is maintained, its maturation is blocked. We obtained similar results using a second myocardial driver (*cTnT*^*Cre*^), but not with an endothelial-specific driver (*Tie2*^*Cre*^), suggesting that Bmp2 needs to reach a certain threshold to drive EMT and prevent cardiomyocyte maturation. Accordingly, forced *Bmp2* expression in vitro stimulated EBs proliferation and blocked their progression into cardiomyogenesis, an effect partially rescued by Noggin. These data demonstrate that Bmp2 is an instructive signal for valve formation and that persistent Bmp2 expression maintains cardiomyocytes in a primitive, proliferative state, which may be relevant for the in vitro expansion of cardiac progenitors for regenerative purposes.

## Results

### Ectopic myocardial *Bmp2* expression disrupts heart morphogenesis

To study the developmental consequences of *Bmp2* expression outside of the valve forming field, we generated a transgenic line in which *Bmp2* expression is activated upon *Cre-*mediated removal of a *β-Geo-stop* cassette (Suppl. Figure S1A and Materials and methods).

**Fig. 1:**
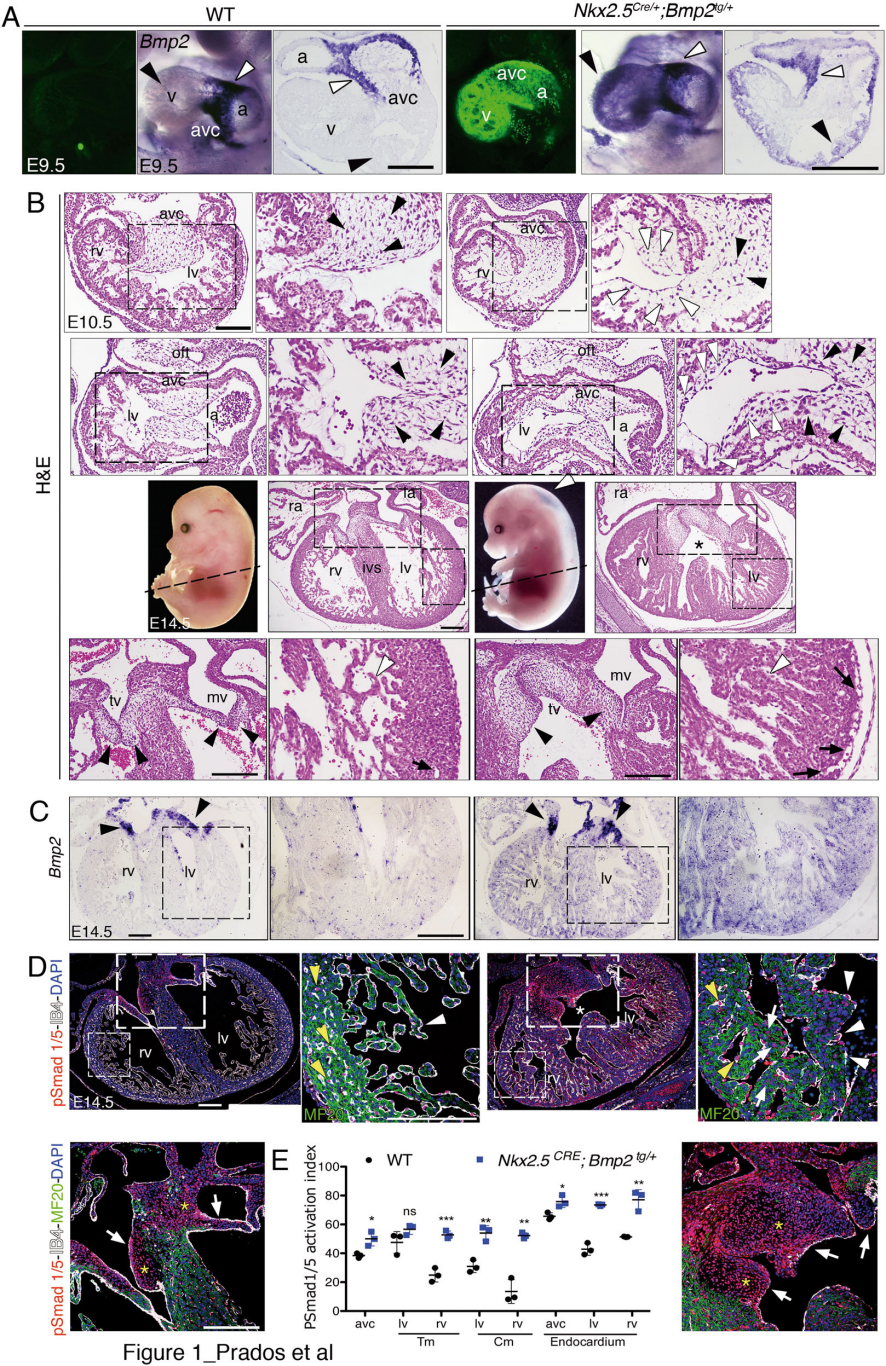
Ectopic myocardial *Bmp2* expression disrupts cardiogenesis. **a** Confocal images of GFP expression plus whole-mount and sectioned in situ hybridisation (ISH) of *Bmp2* in E9.5 WT and *Nkx2*.*5*^*Cre/+*^*;Bmp2*^*tg/+*^ hearts. White arrowheads mark normal *Bmp2* expression in AVC myocardium; black arrowheads mark ectopic *Bmp2* expression in chamber myocardium of the transgenic heart. **b** Top two rows, hematoxylin and eosin (H&E) staining of general and detailed views (insets) of transverse and parasagittal sections of E10.5 WT and *Nkx2*.*5*^*Cre/+*^*;Bmp2*^*tg/+*^ hearts, showing mesenchymal cells in the AVC (black arrowheads) and also in the left ventricles of transgenic hearts (white arrowheads). Third row, whole-mount images of E14.5 WT and *Nkx2*.*5*^*Cre/+*^*;Bmp2*^*tg/+*^ embryos showing oedema in the dorsal region of the transgenic embryo (white arrowhead). H&E stained transverse sections show general and detailed views of ventricular and valve dysmorphology and a ventricular septal defect in the E14.5 *Nkx2*.*5*^*Cre/+*^*;Bmp2*^*tg/+*^ heart (asterisk). Fourth row, details of the atrioventricular (AV) valves (arrowheads) and left ventricle in the WT and transgenic heart; trabeculae (white arrowheads) are larger in the transgenic heart. The arrows mark the WT coronary vessels and the dysmorphic ones in the transgenic heart. **c** ISH showing *Bmp2* mRNA in the AV valves in both E14.5 genotypes (arrowheads) and ectopically throughout the myocardium of *Nkx2*.*5*^*Cre/+*^*;Bmp2*^*tg/+*^ ventricles. **d** Staining for pSmad 1/5 (red), MF 20 (green), isolectin B4 (IB4, white) and DAPI (blue) in E14.5 hearts. General views of transverse heart sections. Note the ventricular septal defect (asterisk) in the transgenic heart. Detailed views show the right ventricle and AV valves of both genotypes, with discrete pSmad 1/5 staining in WT trabecular endocardium (white arrowheads), capillaries in the compact myocardium (yellow arrowheads) and AV valves mesenchyme (arrows, mesenchyme marked with a yellow asterisk). The *Nkx2*.*5*^*Cre/+*^*;Bmp2*^*tg/+*^ heart shows widespread pSmad1/5 expression both in ventricles (including cardiomyocytes, arrows) and in AV valves mesenchyme. **e** pSmad1/5 activation index in the trabecular and compact myocardium (Tm, Cm) of E14.5 WT and transgenic hearts. a atrium, AVC atrioventricular canal, IVS interventricular septum, la left atrium, lv left ventricle, mv mitral valve, ra right atrium, rv right ventricle, tv tricuspid valve, v ventricle; scale bars 200 μm

We expressed *Bmp2* in the developing myocardium using the *Nkx2*.*5*^*Cre*^ line, active since E7.5^12^. At E9.5, transgenic hearts showed widespread GFP expression (Fig. 1a and Suppl. Figure S1A). Whole-mount in situ hybridisation (WISH) showed that *Bmp2* transcription was restricted to AVC myocardium in E9.5 wild type (WT) embryos (Fig. 1a and ref. ^7^), whereas *Nkx2*.*5*^*Cre*^-driven *Bmp2* expression in transgenic embryos was expanded to chamber myocardium (Fig. 1a). Histological analysis of E9.5 WT embryos revealed mesenchyme in AVC cushions and developing ventricular trabeculae (Suppl. Figure S1B). On the contrary, *Nkx2*.*5*^*Cre/+*^*;Bmp2*^*tg/+*^ embryos showed abundant mesenchyme in AVC cushions but also in subendocardium of the right ventricle (Suppl. Figure S1B). This phenotype was more evident at E10.5, when subendocardial mesenchymal cells were observed in AVC and in both ventricles (Fig. 1b). *Nkx2*.*5*^*Cre/+*^*;Bmp2*^*tg/+*^ hearts showed thickened ventricular trabeculae at E12.5 (Suppl. Figure S1B) and at E14.5, embryos were oedematous (Fig. 1b) suggesting cardiovascular deficit. Histological inspection of E14.5 *Nkx2*.*5*^*Cre/+*^*;Bmp2*^*tg/+*^ hearts showed mesenchyme in all the forming valve leaflets, ventricular septal defect, dilated ventricles and coronary vessels, and large trabeculae (Fig. 1b and Suppl. Figure S1B). Transmission electron microscopy revealed denser packing of cardiomyocytes within the trabeculae of E14.5 *Nkx2*.*5*^*CRE/+*^*;Bmp2*^*tg/+*^ embryos (Suppl. Figure S1C, D), as well as poorly defined Z band structure (Suppl. Figure S1C′, C″ versus S1D′, D″), suggesting defective cardiomyocyte maturation.

At E14.5, in situ hybridisation (ISH) showed that *Bmp2* mRNA was restricted to the myocardium of WT AVC (Fig. 1c), whereas in *Nkx2*.*5*^*Cre/+*^;*Bmp2*^*tg/+*^ hearts, *Bmp2* expression was stronger in AVC myocardium and was extended to ventricular myocardium (Fig. 1c). We did not recover alive *Nkx2*.*5*^*Cre/+*^*;Bmp2*^*tg/+*^ embryos beyond E15.5 (Supplemental Table S1). Expression of the phosphorylated Bmp2 signalling effector transcription factors Smad1 and 5 (pSmad1/5)^13^ was moderate in E14.5 WT ventricular endocardium and myocardium, while pSmad1/5 was widely expressed throughout *Nkx2*.*5*^*Cre/+*^*;Bmp2*^*tg/+*^ hearts (Fig. 1d, e). These results demonstrated that *Nkx2*.*5*^*Cre*^-mediated ectopic *Bmp2* expression leads to increased Bmp signalling activity in the myocardium, endocardium and valve mesenchyme.

### Myocardial *Bmp2* expression rescues AVC specification in *Bmp2*-deficient mice

qPCR analysis showed that *Bmp2* expression was moderately elevated in E14.5 *Nkx2*.*5*^*Cre/+*^*;Bmp2*^*tg/+*^ hearts (Suppl. Figure S2A), prompting us to test whether *Nkx2*.*5-Cre*-mediated Bmp2 expression could rescue the AVC specification defect of *Bmp2* mutants. Whole-mount and histological analyses showed that *Nkx2*.*5*^*Cre/+*^*;Bmp2*^*tg/+*^;*Bmp2*^*flox/flox*^ embryos had a well-defined AVC comparable to that of control *Bmp2*^*flox/flox*^ mice (Suppl. Figure S2B), indicating effective rescue of the AVC specification defect of *Nkx2*.*5*^*Cre/+*^*;Bmp2*^*flox/flox*^ mutants. *Bmp2* was expressed in AVC of control, *Nkx2*.*5*^*Cre/+*^*;Bmp2*^*tg/+*^;*Bmp2*^*flox/flox*^ and *Nkx2*.*5*^*Cre/+*^*;Bmp2*^*tg/+*^ embryos, but not in *Nkx2*.*5*^*Cre*^*;Bmp2*^*flox/flox*^ embryos (Suppl. Figure S2B). *Nkx2*.*5*^*Cre*^*;Bmp2*^*flox/flox*^ embryos do not survive beyond E11.5^7,14^, whereas rescued embryos were still alive at E14.5, with defective ventricle and valve development (Suppl. Figure S2C). We found no live *Nkx2*.*5*^*Cre/+*^*;Bmp2*^*tg/+*^;*Bmp2*^*flox/flox*^ embryos beyond E15.5 (Supplemental F S2). These results showed that myocardial expression of *Bmp2* rescues AVC specification of *Bmp2*-deficient mice but leads to lethality at E15.5.

### Ectopic myocardial Bmp2 expression renders the ventricles EMT-competent

**Fig. 2:**
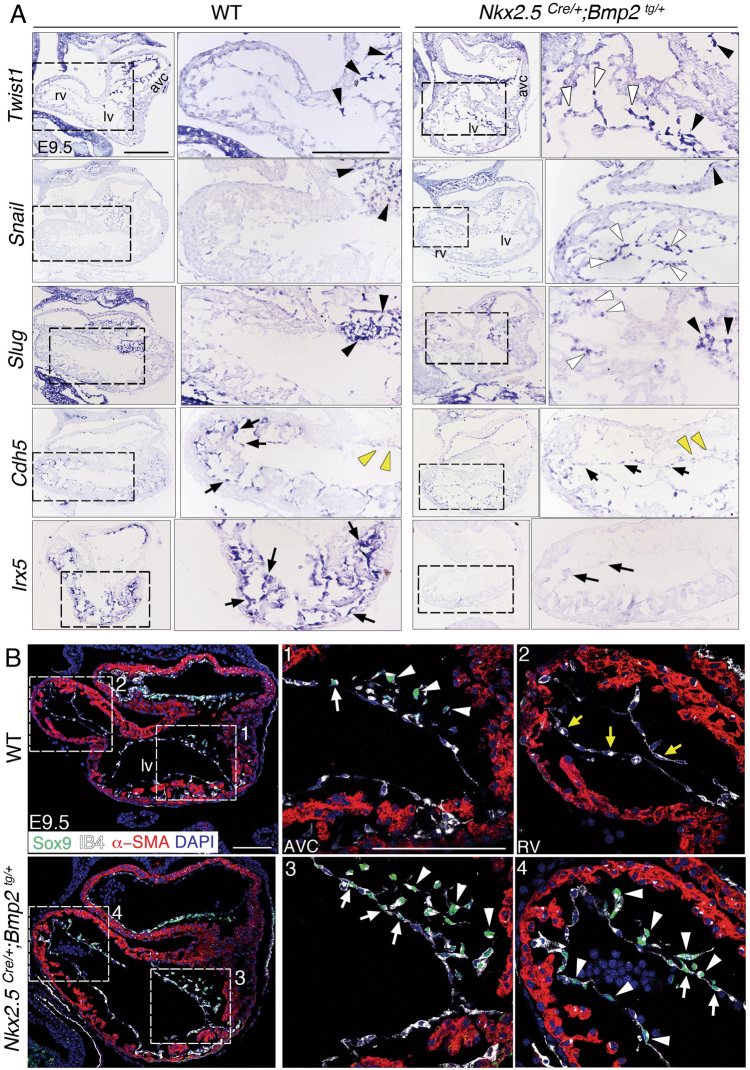
Expanded expression of the EMT drivers *Twist1*, *Snail*, *Slug* and Sox9, and reduction of *Cdh5* and *Irx5* expression in the ventricles of *Nkx2*.*5*^*Cre/+*^*;Bmp2*^*tg/+*^ embryos. **a** ISH showing *Twist1*, *Snail*, *Slug*, *Cdh5* and *Irx5* mRNA in E9.5 WT and *Nkx2*.*5*^*Cre/+*^*;Bmp2*^*tg/+*^ hearts. Black and yellow arrowheads mark expression or lack of expression in AVC mesenchyme cells, respectively; white arrowheads indicate expression in chamber endocardium. *Cdh5* and *Irx5 are* transcribed in WT chamber endocardium, while are strongly reduced in the transgenic ventricle (black arrows). Scale bars 200 μm. **b** Confocal images of E9.5 WT and *Nkx2*.*5*^*Cre/+*^*;Bmp2*^*tg/+*^ hearts stained with Sox9 (green), IB4 (white), α-SMA (red) and DAPI (blue). Mesenchyme (1, arrowheads) and endocardial cells (1, arrow) in the WT AVC express Sox9, while IB4-positive endocardial cells of the right ventricle do not (2, yellow arrows). *Nkx2*.*5*^*Cre/+*^*;Bmp2*^*tg/+*^ hearts show Sox9-positive mesenchyme (3, arrowheads) and endocardial cells (3, arrows) in AVC, but also in the right ventricle (4, arrowheads and arrows). Scale bars 100 μm. Abbreviations as in Fig. 1

**Fig. 3:**
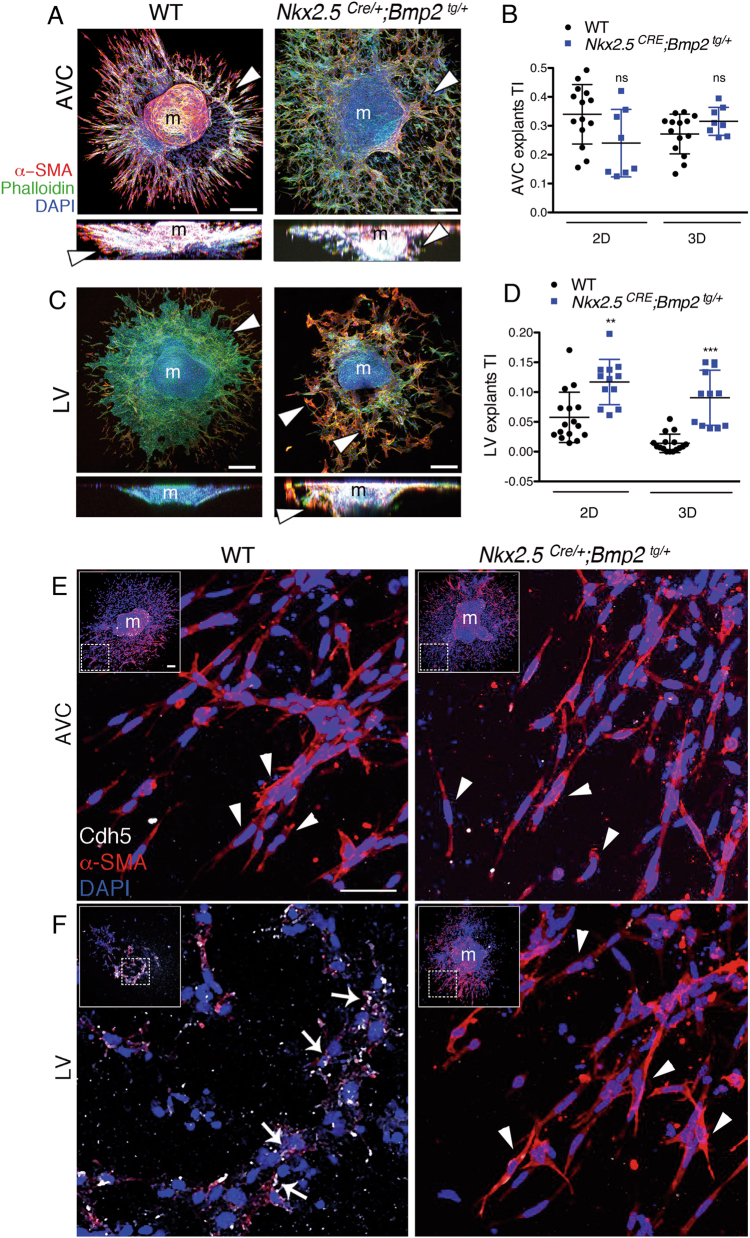
The AVC and ventricles of *Nkx2*.*5*^*Cre/+*^*;Bmp2*^*tg/+*^ embryos undergo EMT in vitro. **a** Confocal images of AVC explants from E9.5 WT and *Nkx2*.*5*^*Cre/+*^*;Bmp2*^*tg/+*^ hearts cultured on collagen gels (top and lateral projections). All explants were stained with phalloidin-FITC (green), anti-α-SMA-Cy3 (red) and DAPI (blue). AVC mesenchymal cells (α-SMA-positive) invade the collagen gel (arrowheads). **b** Quantification of migrating (2D, endocardial) and invading (3D, mesenchyme) cells in WT and transgenic AVC explants. **c** Confocal images of stained left ventricular explants (LV). WT LV endocardial cells do not undergo EMT and form a monolayer on the surface of the gel (arrowhead). Transgenic LV endocardial cells transform, migrate and invade the collagen gel (arrowheads in top views and lateral projections). **d** Quantification of migrating and invading cells in LV explants shows a significantly higher 2D and 3D transformation indexes (TI) in transgenic explants than in WT. TI is the number of migrating (2D) or invading (3D) cells divided by the total number of cells in each explant. m myocardium. *t* test ** *P* < 0.01, ***P < 0.005. **e**, **f** Confocal images of explants. **e** The insets show general views of WT and *Nkx2*.*5*^*Cre/+*^*;Bmp2*^*tg/+*^ AVC explants stained with anti-Cdh5 (white), anti-α-SMA (red) and DAPI (blue). The large images are a magnification of the area marked in the insets and show mesenchymal cells stained with anti-α-SMA and DAPI, but not with anti-Cdh5 (arrowheads). **f** The insets show general views of WT and *Nkx2*.*5*^*Cre/+*^*;Bmp2*^*tg/+*^ ventricular explants stained with the same antibodies than above (in the WT ventricular explant the myocardium (m) has been removed). The large images are a magnification of the area marked in the insets and show that WT ventricular endocardial cells express Cdh5 (F, white arrows), while transformed mesenchymal cells in transgenic ventricular explants express α-SMA (F, arrowheads). Scale bars 100 μm in insets; 50 μm in large images

**Fig. 4:**
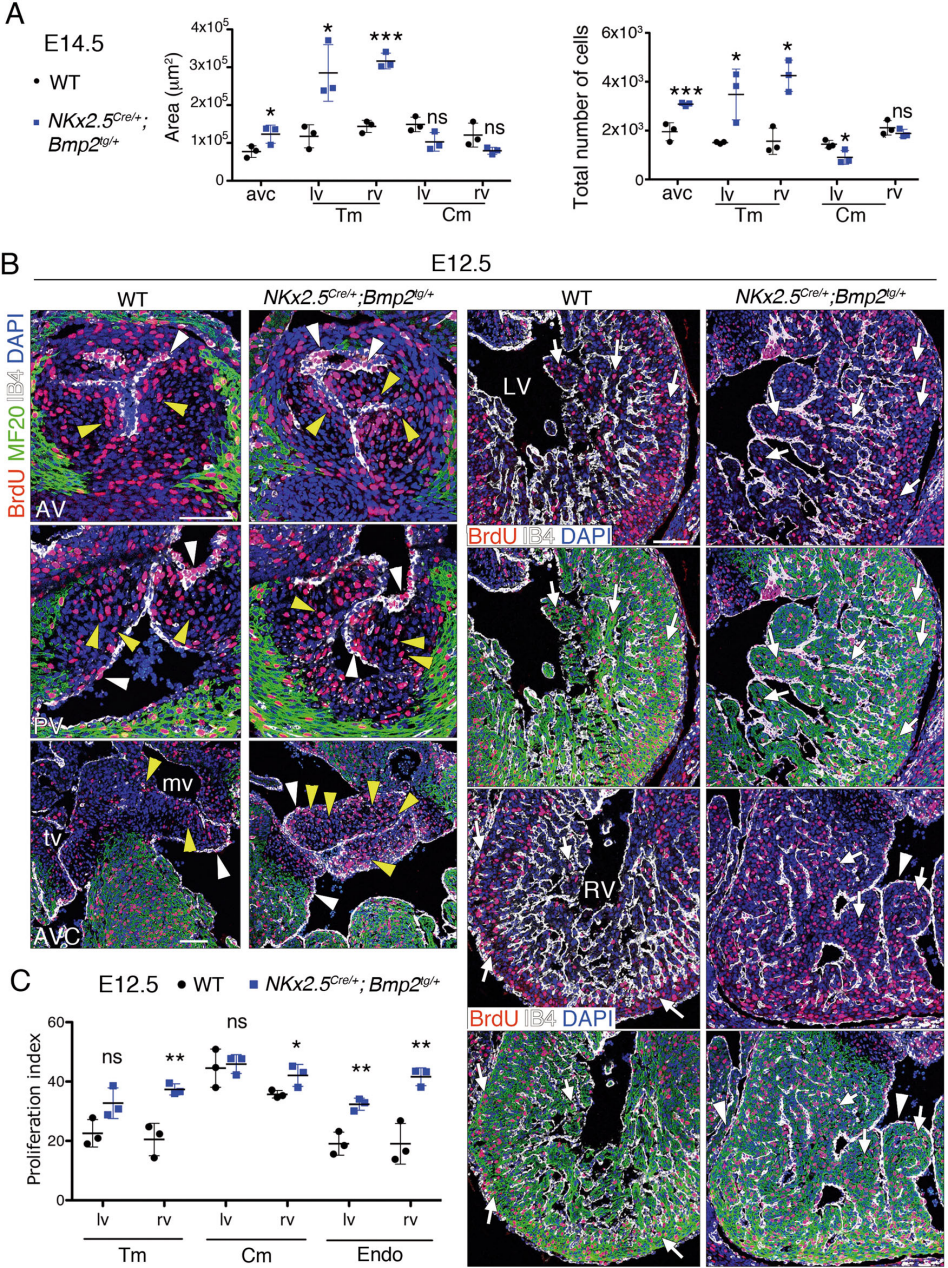
*Nkx2*.*5*^*Cre/+*^*;Bmp2*^*tg/+*^ embryos show increased cardiac proliferation. **a** Area occupied by the various cardiac territories, and the total number of cells within them, analysed in E14.5 WT and *Nkx2*.*5*^*Cre/+*^*;Bmp2*^*tg/+*^ hearts. **b** Proliferation analysis in E12.5 WT and transgenic heart sections. Sections are stained with an anti-BrdU (red) and anti-MF20 (green, myocardium) antibodies, isolectin B4 (white, endocardium) and counterstained with DAPI (blue). AV aortic valve, AVC atrioventricular valves, LV left ventricle, mv mitral valve, RV right ventricle, tv tricuspid valve. The top rows in the column corresponding to the sections of the ventricles show only anti-BrdU, IB4 and DAPI staining, to facilitate identification of BrdU-positive nuclei. The white and yellow arrowheads indicate BrdU-positive endocardium or mesenchyme nuclei, respectively. The white arrows indicate BrdU-positive nuclei in myocardium. Scale bars 200 μm. **c** Proliferation index in E12.5 WT and transgenic hearts is the ratio of BrdU-positive nuclei to the total number of nuclei in each cell type. *t* test, *P < 0.05; **P < 0.01; ns non-significant

The cardiac EMT drivers Twist1^7^, Snail^15^ and Slug^16^ are transcribed in the WT AVC endocardium and mesenchyme, and their expression was expanded to the ventricles in E9.5 *Nkx2*.*5*^*Cre/+*^*;Bmp2*^*tg/+*^ embryos (Fig. 2a). Snail represses *vascular endothelial cadherin* (*Cdh5*) expression in E9.5 AVC endocardium so that EMT occurs^15^. ISH confirmed absence of *Cdh5* expression in WT AVC endocardium, and strong expression in ventricular endocardium (Fig. 2a). In contrast, E9.5 *Nkx2*.*5*^*Cre/+*^*;Bmp2*^*tg/+*^ embryos showed weaker and discontinuous *Cdh5* expression in ventricular endocardium (Fig. 2a). E9.5 *Nkx2*.*5*^*Cre/+*^*;Bmp2*^*tg/+*^ embryos also had severely reduced expression of the chamber endocardium marker *Irx5*^17^ (Fig. 2a), indicating loss of chamber endocardium identity. qPCR analysis confirmed the increased expression of the EMT drivers *Tgfβ2*, *Snail1* and *Twist1* in E9.5 *Nkx2*.*5*^*Cre/+*^*;Bmp2*^*tg/+*^ hearts (Suppl. Figure S2D). We also immunostained E9.5 embryos with Sox9, a well-known Bmp2 target in EMT^7,18^, isolectin B4 (IB4) to label the endocardium and α-smooth muscle actin (α-SMA) to mark the myocardium. In the E9.5 WT heart, Sox9 is expressed in AVC mesenchymal cells (Fig. 2b-1, 2), whereas in *Nkx2*.*5*^*Cre/+*^*;Bmp2*^*tg/+*^ embryos Sox9 is expressed in AVC endocardium and mesenchyme, but also in ventricular endocardium (and derived mesenchymal cells, Fig. 2b-3, 4). These observations indicate that myocardial Bmp2 expression leads to ectopic EMT drivers activation, that will induce transformation in ventricular endocardial cells.

At E14.5, these EMT markers were still upregulated (Suppl. Figure 2A) although expression was confined to the enlarged valves (Suppl. Figure S3A).

We assayed in explants the ability of myocardial *Bmp2* to induce ectopic EMT. We first explanted AVC tissue from WT and transgenic hearts as described^1,15^ and measured the two-dimensional (2D) and three-dimensional (3D) transformation index (TI), which define the migratory and invasive capacity of the explants, respectively^19^. E9.5 WT and *Nkx2*.*5*^*Cre/+*^*;Bmp2*^*tg/+*^ AVC explants generated mesenchymal cells invading the collagen gel (Fig. 3a). The 2D and 3D TI values were similar for both genotypes (Fig. 3a, b). Then, we carried out explants with the left ventricle. Cultures from WT ventricles generated a coherent endocardial monolayer surrounding the myocardium (Fig. 3c). In contrast, *Nkx2*.*5*^*Cre/+*^*;Bmp2*^*tg/+*^ ventricular explants gave rise to mesenchymal cells that invaded the collagen matrix, similarly to control AVC explants (Fig. 3c, d). These ventricular endocardium-transformed mesenchymal cells had a significantly higher 2D TI and 3D TI than WT explants (Fig. 3c, d). We stained with anti-Cdh5 and α-SMA antibodies, to label the various cell types in the explants^15^. Figure 3e shows that transformed mesenchymal cells in both WT and transgenic AVC explants stain with α-SMA, but not with anti-Cdh5. Endocardial cells of WT ventricular explants, that do not transform (Fig. 3c, d), express Cdh5 and do not express α-SMA (Fig. 3f, left). In contrast, transformed mesenchymal cells of *Nkx2*.*5*^*Cre/+*^*;Bmp2*^*tg/+*^ ventricular explants express α-SMA, but not Cdh5 (Fig. 3f, right).

### Myocardial *Bmp2* gain-of-function stimulates cardiomyocyte proliferation and prevents chamber maturation

The enlarged trabeculae and valves in E14.5, *Nkx2*.*5*^*Cre/+*^*;Bmp2*^*tg/+*^ embryos (Fig. 1b) prompted us to measure the surface of the myocardium and AVC, and their total number of cells. The compact myocardium area was similar in WT and transgenic embryos, whereas the trabecular myocardium and AVC regions were larger and contained more cells in E14.5 *Nkx2*.*5*^*Cre/+*^*;Bmp2*^*tg/+*^ embryos (Fig. 4a).

We examined cell proliferation by measuring 5-bromodeoxyuridine (BrdU) incorporation in WT and *Nkx2*.*5*^*Cre/+*^*;Bmp2*^*tg/+*^ E12.5 hearts. We observed a significantly increased proliferation in AVC valves mesenchyme, but not in aortic or pulmonary valves of transgenic hearts (Suppl. Figure S3C and Fig. 4b). Proliferation was also increased in compact and trabecular myocardium of the right ventricle, and throughout ventricular endocardium (Fig. 4b, c). This elevated proliferation was sustained but less pronounced at E14.5 (Suppl. Figure S3B, C). Thus, *Bmp2* overexpression in *Nkx2*.*5*^*Cre/+*^*;Bmp2*^*tg/+*^ embryos leads to a proliferative expansion of valves and trabeculae.

**Fig. 5:**
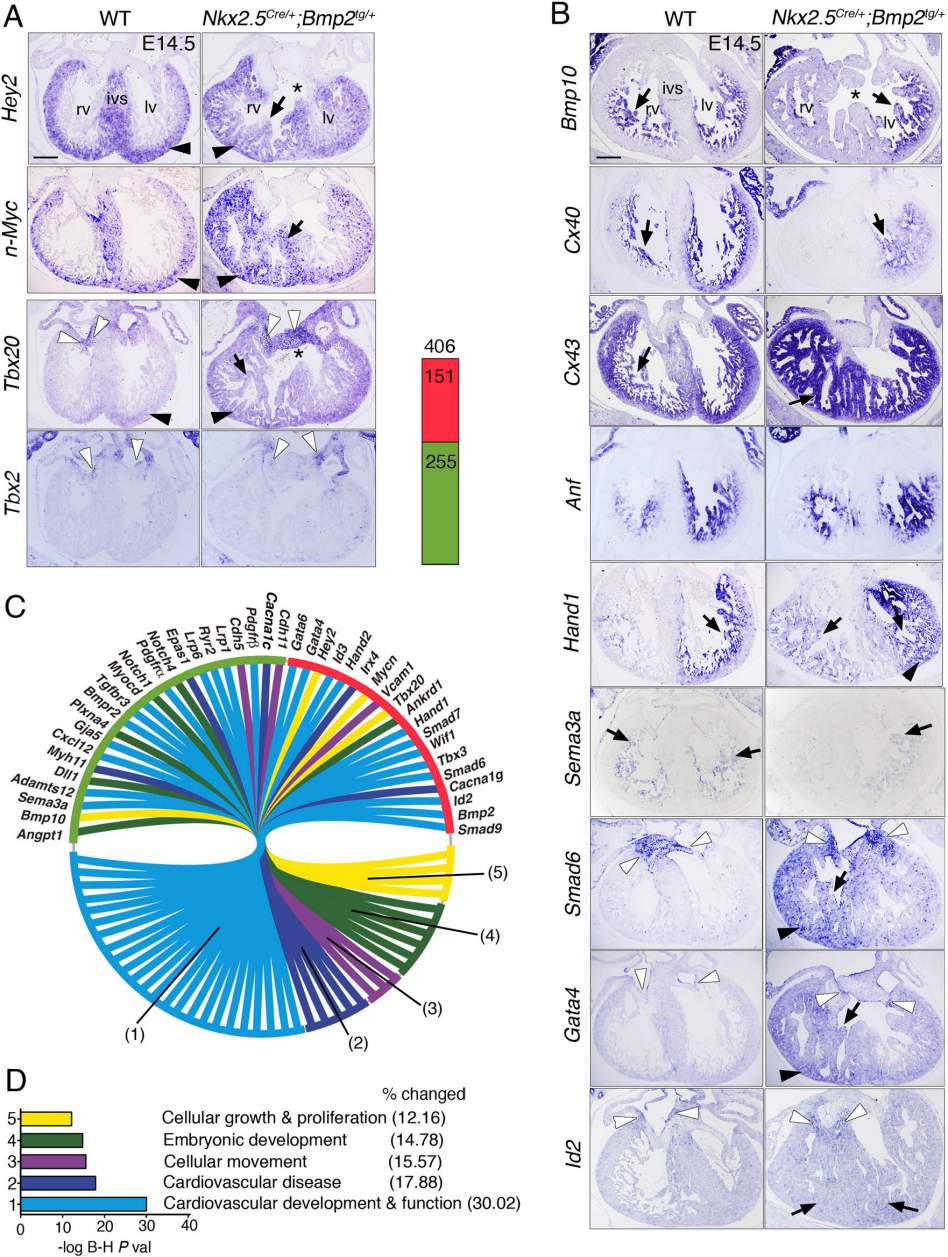
*Nkx2*.*5*^*Cre/+*^*;Bmp2*^*tg/+*^ embryos show defective ventricular chamber maturation. **a** E14.5, ISH. The black arrowheads mark compact myocardium, arrows mark trabeculae, white arrowheads the AVC valves region and the asterisks indicate a ventricular septal defect. ISH of *Hey2*, *n-Myc*, *Tbx20* and *Tbx2* in E14.5 WT and *Nkx2*.*5*^*Cre/+*^*;Bmp2*^*tg/+*^ hearts. **b** ISH of *Bmp10*, *Cx40*, *Cx43*, *Anf*, *Hand1*, *Sema3a*, *Smad6*, *Gata4* and *Id2* in E14.5 WT and transgenic hearts. IVS interventricular septum, rv right ventricle, lv left ventricle. Scale bars 200 μm. **c** Left, Circular plot of 42 differentially expressed genes in E14.5 transgenic hearts. Top, Chart showing the total number of upregulated (red) and downregulated genes (green). Details are available in Supplementary file 1. **d** B-H *P* value, Benjamini–Hochberg-adjusted *P* value. % changed classifies the genes altered in *Nkx2*.*5*^*Cre/+*^*;Bmp2*^*tg/+*^ transgenic hearts as a percentage of the total number of genes involved in the listed biological function. *t* test *P < 0.05; **P < 0.01; ***P < 0.005

The compact myocardium markers *Hey2*^20^ and *n-Myc*^21^ were expanded to trabecular myocardium at E14.5 (Fig. 5a), suggesting impaired chamber maturation in transgenic embryos. Likewise, *Nkx2*.*5*^*Cre/+*^*;Bmp2*^*tg/+*^ embryos showed increased *Tbx20* expression in AVC valves and expansion to the trabeculae (Fig. 5a). In contrast, *Tbx2* was normally expressed in AVC valves (Fig. 5a). These results are in accordance with the reported repression of *Tbx2* by Hey2 and Tbx20 in chamber myocardium, resulting in AVC-restricted *Tbx2* expression^10,22^. Tbx20 interacts with Bmp/Smad signalling to confine *Tbx2* expression to the AVC^23^.

The trabecular markers *Bmp10*^24^ and *Cx40/Gja5*^25^ were downregulated in *Nkx2*.*5*^*Cre/+*^*;Bmp2*^*tg/+*^ embryos (Fig. 5b), while *Cx43* was upregulated and *Anf* unaffected (Fig. 5b). The transcription factor Hand1, a Tbx20 effector^26,27^ was upregulated and extended to the right ventricle of transgenic embryos (Fig. 5b). These results, together with the expansion of the compact myocardium markers (Fig. 5a), and the increased cardiomyocyte proliferation in *Nkx2*.*5*^*Cre/+*^*;Bmp2*^*tg/+*^ embryos (Fig. 4b, c and Suppl. Figure S3B, C), indicate that ectopic myocardial *Bmp2* expression disrupts ventricular chamber maturation by promoting proliferation and preventing cardiomyocyte differentiation.

### Gene profiling reveals dysregulated cardiovascular development and cellular proliferation

RNA sequencing (RNA-seq) identified altered cardiac expression of 406 genes (*P* ≤ 0.05) in E14.5 *Nkx2*.*5*^*Cre/+*^*;Bmp2*^*tg/+*^ hearts, 151 genes were upregulated and 255 downregulated (Fig. 5c and Suppl. File S1). Confirming our ISH analysis, *Bmp2*, *Tbx20*, *n-Myc*, *Hey2*, *Hand1* and *Hand2* were upregulated, whereas *Cx40* and *Bmp10* were downregulated (Fig. 5c and Suppl. File S1). Gene ontology (GO) classification revealed that most dysregulated genes in *Nkx2*.*5*^*Cre/+*^*;Bmp2*^*tg/+*^ hearts were involved in cardiac development and disease, and cell growth and proliferation (Fig. 5c, d and Supplemental File 1). Genes implicated in coronary vessel formation (*Hey1*, *Dll4*, *Epas1*, *Sema3a*, *Angpt1*) were also downregulated (Fig. 5c). ISH for the coronary artery markers *Dll4*, *Hey1*, *Cxcr4* and *Fabp4*^28^ confirmed these data (Fig. 5b and Suppl. Figure S4A–H). The number of *Fabp4*-positive vessels was reduced in E14.5 *Nkx2*.*5*^*Cre/+*^*;Bmp2*^*tg/+*^ embryos (Suppl. Figure S4I), indicating defective coronary vessel development.

*Nkx2*.*5*^*Cre/+*^*;Bmp2*^*tg/+*^ embryos also showed increased *Smad6* and *Smad7* expression (Fig. 5c), which encode inhibitory Smads^29^. *Smad6* was ectopically expressed in E14.5 *Nkx2*.*5*^*Cre/+*^*;Bmp2*^*tg/+*^ ventricles (Fig. 5b), presumably reflecting a negative-feedback loop triggered by ectopic Bmp2 expression, as in vitro experiments suggested^30^. Similarly, the cardiac specification marker *Gata4*^31^ was upregulated in myocardium, especially of the right ventricle in *Nkx2*.*5*^*Cre/+*^*;Bmp2*^*tg/+*^ embryos, and in AVC valves endocardium (Fig. 5b). RNA-seq (Fig. 5c) and qPCR data (Suppl. Figure S2A) showed upregulation of *Id2*, a n-Myc effector critical for cell proliferation^32^. *n-Myc* is a direct downstream target of Smad4^33^. In addition, both Hey2 and n-Myc are required for cardiomyocyte proliferation^20,34^, suggesting that the increased cardiac proliferation observed in *Nkx2*.*5*^*CRE/+*^*;Bmp2*^*tg/+*^ embryos (Fig. 4b, c) could be mediated by *Hey2*, *n-Myc* and *Id2* activation downstream of Bmp2.

### *Bmp2* gain-of-function in myocardium, but not endothelium, disrupts ventricular development

We used the myocardial-specific *cTnT-Cre* line, active from E8.0 onwards^35^, that caused a phenotype similar to *Nkx2*.*5*^*Cre/+*^*;Bmp2*^*tg/+*^ embryos. E10.5 c*TnT*^*Cre/+*^*;Bmp2*^*tg/+*^ mice showed enlarged AVC, and mesenchymal cells in the right ventricle (Suppl. Figure S5A). E14.5 c*TnT*^*Cre/+*^*;Bmp2*^*tg/+*^ embryos had ventricular septal defect, dilated ventricles and coronaries, and thickened valves and trabeculae (Suppl. Figure S5B), and did not progress beyond E17.5 (Supplemental Table S3). c*TnT*^*Cre/+*^*;Bmp2*^*tg/+*^ hearts expressed *Bmp2* throughout the myocardium (Suppl. Figure S5B). These embryos showed upregulated *Tbx20* expression (Suppl. Figure S5C), expansion of *Hey2* to the trabeculae (Suppl. Figure S5C) and reduced *Cx40* and *Bmp10* expression (Suppl. Figure S5D), indicating impaired ventricular chamber maturation. Coronary vessels were also affected, as the loss of *Cx40* expression indicated (Suppl. Figure S5D). *Tbx2* and *Cx43/Gja1* expression was unaltered (Suppl. Figure S5D).

Myocardium-derived Bmp2 binds to its receptor in AVC endocardium to activate EMT^7^. We used the *Tie2*^*Cre*^ driver to express Bmp2 in vascular endothelium and endocardium from E7.5^36^. E10.5 *Tie2*^*Cre/+*^*;Bmp2*^*tg/+*^ embryos had normal heart morphology (Suppl. Figure S5E and data not shown). Indeed, *Tie2*^*Cre/+*^*;Bmp2*^*tg/+*^ embryos developed normally and reached adulthood (Supplemental Table S4). qPCR showed a slight increase in *Bmp2* transcription and normal *Twist1*, *Snail*, *Cdh5* and *Slug* expression in E14.5 transgenic embryos (Suppl. Figure S6A). These results indicate that Tie2-Cre-mediated ectopic *Bmp2* expression does not affect cardiogenesis, and indicate that the phenotypic similarities between *Nkx2*.*5*^*Cre/+*^*;Bmp2*^*tg/+*^ and *cTnT*^*Cre/+*^*;Bmp2*^*tg/+*^ mice are due to myocardial *Bmp2* overexpression.

### Bmp2 stimulates proliferation and prevents cardiomyogenesis in vitro

**Fig. 6:**
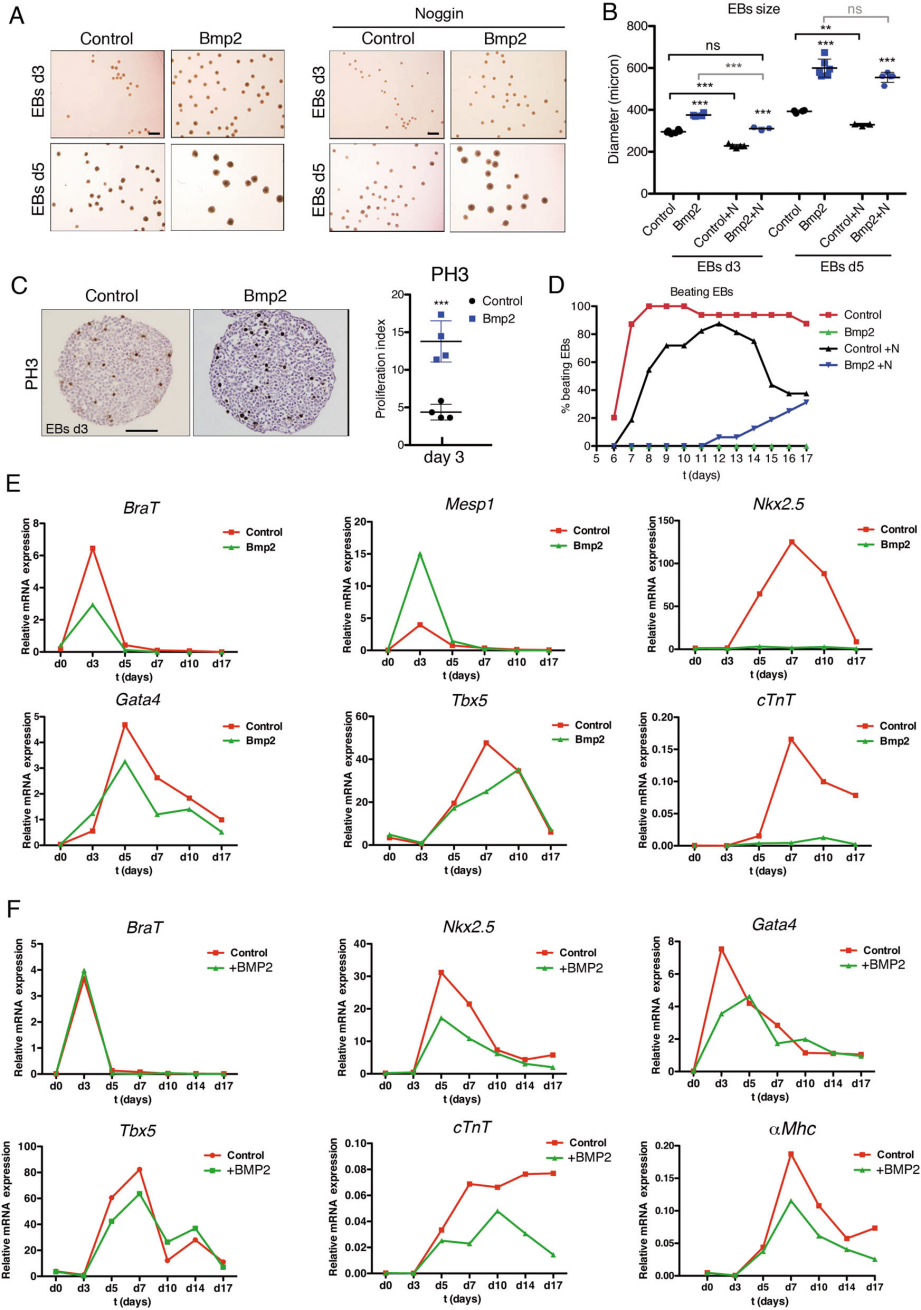
Constitutive Bmp2 expression leads to increased proliferation and blockade of cardiac differentiation in embryoid bodies (EBs). **a** Images showing EBs size at day 3 (d3) and day 5 (d5) of culture. Bmp2-expressing-EBs (Bmp2) are visibly larger than controls. Noggin (500 ng/ml, right panels) reduced the size of both control and Bmp2-EBs at d3 but not the size of Bmp2-EBs at d5. Scale bars 1 mm. **b** Size quantification of the EBs shown in **a** (control, WT EBs; Bmp2, *Bmp2*-EBs;+N, Noggin-treated EBs). Bmp2-EBs were larger than WT EBs at d3 and d5 (Bmp2-EBs vs. Control EBs). Noggin significantly reduced the size of control and Bmp2-EBs at d3 but not of d5 Bmp2-EBs at d5 (treated EBs vs untreated EBs). Noggin-treated Bmp2-EBs were similar in size to untreated WT EBs at d3 but not at d5. ***P < 0.005, **P < 0.01, ns, non-significant one-way ANOVA (non-parametric) and Bonferroni post-test. **c** Phospho-Histone3 (PH3) staining in d3 control and Bmp2-EBs. Quantification shows a significant increase in PH3-positive nuclei. Scale bar 200 μm. *t*-test, ***P < 0.005. **d** Beating ability of WT EBs in the absence (red) or presence of Noggin (500 ng/ml) (black), or Bmp2-EBs in the absence (green) or presence of Noggin (blue). **e**, **f** Gene expression (qRT-PCR) of cardiac specification markers (*BraT* and *Mesp1*), early cardiac differentiation markers (*Nkx2*.*5*, *Gata4*, *Tbx5* and *cTnT*) and late differentiation marker (*αMhc*) in **e** WT EBs (Control, red) and Bmp2-EBs (Bmp2, green) and **f** WT EBs (Control, red) and human recombinant BMP2-treated EBs (+BMP2, green)

The increased proliferation and impaired cardiomyocyte maturation of *Nkx2*.*5*^*Cre/+*^*;Bmp2*^*tg/+*^ mice prompted us to test the ability of *R26CAGBmp2-eGFP* mouse embryonic stem cells (mESC) to form embryoid bodies (EBs) and differentiate into cardiomyocytes^37^. *R26CAGBmp2-eGFP* mESCs were transfected with a Cre-expressing plasmid, and GFP-positive clones (Bmp2-mESCs) were identified (Suppl. Figure S6B). Non-recombined clones were used as control mESCs. ELISA revealed around 3.5-fold Bmp2 increase in the culture medium of Bmp2-ESCs (Suppl. Figure S6C). Bmp2-expressing embryoid bodies (Bmp2-EBs) expressed GFP, whether cultured in suspension or plated (Suppl. Figure S6D). Bmp2-EBs at day 3 (d3) and d5 of culture were larger than control EBs (Fig. 6a, b). To determine whether this size difference was due to Bmp2 overexpression, we cultured EBs with different concentrations (see Experimental procedures) of the Bmp antagonist Noggin^38^. Overall, 500 ng/ml Noggin had an effect on Bmp2-EBs, being added from 3 days before EBs formation (d-3) to day 17 (d17). Noggin reduced the size of control and Bmp2-EBs at d3 (Fig. 6a,b) and prevented Bmp2-EBs from growing more than control EBs (Fig. 6a, b). By d5, Noggin was unable to reduce the size of Bmp2-EBs (Fig. 6a, b), but reduced the size of control EBs (Fig. 6a, b). Phospho-Histone3 (PH3) and BrdU analyses revealed markedly increased proliferation in Bmp2-EBs at d3 (Fig. 6c and Suppl. Figure S6E). These data indicate that Bmp2 overexpression in EBs promotes proliferation and that continuous Noggin-mediated Bmp2 blockade limits this effect.

We examined the beating ability of Bmp2-EBs from d6-d17 as an indication of cardiomyocyte differentiation^37^. Control EBs started beating at day 6, with more than 90% beating from d8-d17; in contrast, Bmp2-EBs did not beat at any time-point assayed (Fig. 6d). Noggin (500 ng/ml) partially restored beating ability in Bmp2-EBs 14 days after administration, with 30% of the culture beating by d17 (Fig. 6d). qPCR revealed an increase in *Bmp2* and *GFP* expression in Bmp2-EBs (Suppl. Figure S6E). The d3 expression spike of the early mesodermal marker *Brachyury* (*BraT*)^39^ was suppressed in Bmp2-EBs, whereas the cardiac mesoderm marker *Mesp1*^40^ was upregulated (Fig. 6e). The cardiac progenitor marker *Nkx2*.*5*^41^ was undetectable, whereas the *Gata4* and *Tbx5* responses were decreased (Fig. 6e). Bmp2-EBs showed no detectable expression of the early cardiomyocyte differentiation marker *cardiac Troponin T* (*cTnT*) (Fig. 6e), and several late markers (Suppl. Figure S6F), suggesting that Bmp2-EBs do not progress to cardiomyogenesis. Similarly to the in vivo situation (Fig. 5b, c), *Smad6* transcription was increased in Bmp2-EBs (Suppl. Figure S6F).

Our in vitro data show that constitutive Bmp2 overexpression in EBs affects cardiac differentiation. To test whether the increased BMP2 concentration affected cardiogenesis after cardiac specification had taken place, we cultured control *R26CAG-Bmp2* mESCs in the presence or absence of human BMP2 (20 ng/ml) from d3 to d17. The beating ability of WT and BMP2-treated EBs was similar. qPCR on samples collected before and after BMP2 addition revealed no differences in *BraT* or *Mesp1* expression between BMP2-treated and untreated EBs (Fig. 6f and Suppl. Figure S6G); however, BMP2 addition decreased the expression of early (Fig. 6f) and late cardiac differentiation markers (Suppl. Figure S6G). This result is consistent with our in vivo results and previous in vitro data^11^ suggesting that increased BMP2 signalling following cardiac mesoderm specification prevents cardiomyocyte differentiation.

## Discussion

By activating *Bmp2* expression throughout the embryonic myocardium, we provide genetic evidence indicating that Bmp2 is an instructive myocardial signal, able to induce the formation of cardiac valve primordia from AVC and non-AVC endocardial cells. Bmp2 gain in the embryonic myocardium promotes cardiac cell proliferation, disrupts valve remodelling and chamber cardiomyocyte patterning and maturation. Our data are informative about the mechanisms underlying mammalian cardiac patterning, and suggest that timely Bmp2 activation may be useful in the ex vivo expansion of immature cardiomyocytes.

### Valve primordium formation, Bmp2 and endocardial competence

Explant assays with chicken AVC and ventricles showed that only AVC endocardium was able to undergo EMT^1^. Expression, explants and loss-of-function studies in mice confirmed that Bmp2 is crucial for AVC specification and cushion formation^5–8^. Our results show that myocardial Bmp2 gain directs ectopic EMT, expansion of EMT markers to ventricles and loss of chamber endocardial identity. Our data overturn the notion that only AVC endocardial cells are “competent” to respond to Bmp2^1^, as ventricular endocardial cells also respond to Bmp2, upregulate EMT drivers and undergo full transformation. Thus, AVC endocardium competence to undergo EMT results from the tightly regulated AVC-restricted Bmp2 signalling, and not from the segregation of EMT-competent and non-competent endocardial cells in the early embryo.

The inability of forced endothelial-endocardial Bmp2 expression to trigger ectopic EMT, and the slight increase in *Bmp2* transcription, suggests that *Tie2*^*Cre*^-driven Bmp2 levels might not reach a threshold required for promoting EMT outside the AVC. Our results show that forced myocardial Bmp2 expression promotes ectopic EMT, but does not alter the timing of valve primordium formation, as indicates the normal EMT drivers expression in the valves of E14.5 *Nkx2*.*5*^*Cre/+*^*;Bmp2*^*tg/+*^ embryos.

Bmp2 has been suggested to regulate AVC myocardial patterning through *Tbx2* activation^7^. Hey2 and Tbx20 both directly repress *Tbx2* in chamber myocardium, and confine its expression to the AVC through interaction with Bmp/Smad signalling^10,22,23^. Thus, the expanded expression of *Hey2* and *Tbx20* in E14.5 *Nkx2*.*5*^*Cre/+*^*;Bmp2*^*tg/+*^ embryos may explain why *Tbx2* expression does not extend to the ventricles, despite being positively regulated by Bmp2^42^. In addition, *Bmp10* induces *Tbx20* promoter activity in vitro through a conserved Smad1 binding site^43^. We hypothesise that the expanded Smad1/5 expression in *Nkx2*.*5*^*Cre/+*^*;Bmp2*^*tg/+*^ embryos could induce *Tbx20* expression in a Smad-dependent manner, and thus repress *Tbx2* in the chambers.

### Proliferative and differentiation-inhibitory effects of Bmp2 gain in the myocardium

**Fig. 7:**
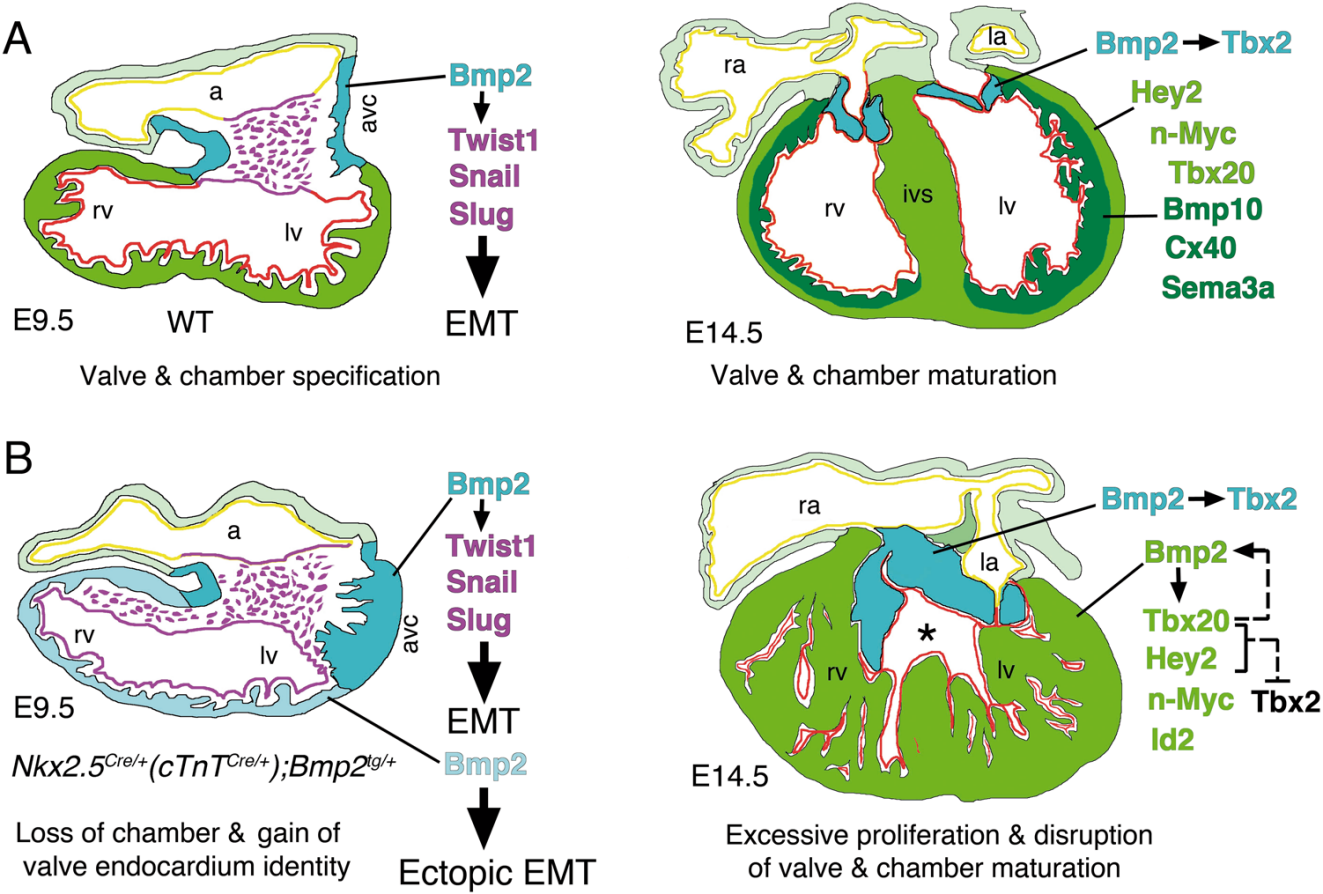
Myocardial Bmp2 gain promotes ectopic EMT, stimulates cardiac proliferation and disrupts ventricular patterning and cardiomyocyte maturation. **a** Left, E9.5 WT heart, chamber (ventricles and atria, green and light green) and non-chamber myocardium (AVC, blue) are specified. Bmp2 expression in AVC myocardium drives EMT via *Twist1*, *Snail* and *Slug* activation in AVC endocardium (purple). The Irx5-positive ventricular endocardium is coloured in red and the atrial endocardium in yellow. Right, E14.5 WT heart, the compacting ventricular myocardium is formed by an outer compact myocardium (Hey2-, n-Myc-positive, light green) and an inner trabecular myocardium (Bmp10-, Cx40-, Sema3a-positive, dark green). Tbx20 is expressed in compact and weakly, in trabecular myocardium. The maturing AVC valves (blue) express Bmp2 and Tbx2. B; left, E9.5 *Nkx2*.*5*^*Cre/+*^(or *cTnT*^*Cre/+*^)*;Bmp2*^*tg/+*^ transgenic heart. Bmp2 is normally expressed in AVC myocardium (blue) and ectopically in ventricular myocardium (light blue), driving Twist1, Snail and Slug expression in the Irx5-negative ventricular endocardium (purple), causing ectopic EMT. *Right*, E14.5 *Nkx2*.*5*^*Cre/+*^(or *cTnT*^*Cre/+*^)*;Bmp2*^*tg/+*^ transgenic heart. Bmp2 is expressed in an expanded AVC valve region (blue) and throughout the ventricles (green), leading to increased and expanded *Tbx20*, *Hey2*, *n-Myc* and *Id2* expression in this tissue. As a consequence, cardiomyocyte proliferation is increased and chamber patterning/maturation is disrupted. The discontinuous arrow represents a potential positive feedback loop between Tbx20 and Bmp2 and the suggested negative regulation of Tbx2 by Tbx20 and Hey2. The asterisk indicates a ventricular septal defect. Abbreviations as in Fig. 1

The increased cellular proliferation in *Nkx2*.*5*^*Cre/+*^*;Bmp2*^*tg/+*^ hearts could seem paradoxical, since Bmp2 is normally expressed in the AVC, which is less proliferative than chamber myocardium^44^. Thus, one would expect that myocardial Bmp2 overexpression would attenuate cardiomyocyte proliferation. However, myocardial Bmp2 overexpression leads to increased and expanded expression of genes promoting cardiomyocyte proliferation, such as *Hey2*, *n-Myc*, *Id2* and *Tbx20*^20,21,32,33^ (Fig. 7). Myocardial Bmp2 overexpression causes a phenotype similar to that caused by myocardial Tbx20 overexpression, which through BMP2/pSmad1/5/8 signalling promotes cardiomyocyte proliferation and maintenance of embryonic characteristics in foetal and adult mouse hearts^45,46^. Therefore, the expanded *Tbx20* expression in *Nkx2*.*5*^*Cre/+*^*;Bmp2*^*tg/+*^ embryos may reflect a positive feedback loop by which the expanded Smad1/5 expression in *Nkx2*.*5*^*Cre/+*^*;Bmp2*^*tg/+*^ embryos could induce *Tbx20* expression throughout the heart. *Smad4* inactivation with *cTnT*^*Cre*^ leads to a hypocellular myocardial wall, due to reduced ventricular cardiomyocyte proliferation. Expression of the Smad4 target *n-Myc* is downregulated in myocardial *Smad4* mutants, as well as that of n-Myc target genes *Cyclin D1*, *D2* and *Id2*^33^. This negative effect of Bmp signalling loss on cardiomyocyte proliferation is compatible with our data showing the positive effect on cardiomyocyte proliferation of myocardial Bmp2 gain.

Structural, marker and gene profiling analyses revealed that increased myocardial proliferation in *Nkx2*.*5*^*Cre/+*^*;Bmp2*^*tg/+*^ embryos is accompanied by defective ventricular chamber maturation, with an expansion of the more primitive and proliferative compact myocardium markers and the downregulation of trabecular markers (Fig. 7). These in vivo data were consistent with our in vitro EB differentiation results showing that constitutive Bmp2 expression stimulates EB proliferation, attenuates cardiac mesoderm specification and prevents cardiomyocyte differentiation. Defective maturation of Bmp2-EBs is reflected in the lack of beating ability, and reduced expression of *Nkx2*.*5*, *Gata4* and *Tbx5*. Addition of BMP2 to the medium after cardiac specification did not affect EB beating but did impair cardiac differentiation. These observations are consistent with those showing that transient BMP signalling inhibition induces cardiomyocyte differentiation of mESC^11^. Thus, our data show that widespread myocardial Bmp2 expression maintains chamber myocardium and early cardiac progenitors in a primitive, proliferative state and identify Bmp2 as a potential factor for the expansion of cardiomyocytes in vitro. Studies in zebrafish show that *bmp2b* overexpression stimulates cardiomyocyte dedifferentiation and proliferation and enhance cardiac regeneration^47^. Likewise, *Tbx20* overexpression in adult cardiomyocytes promotes their proliferation and improves cardiac function after myocardial infarction through the activation of multiple pro-proliferation pathways, including BMP signalling^48^.

## Materials and methods

### Generation of *R26CAGBmp2* transgenic line

See the supplemental experimental procedures.

### Additional mouse lines

The following mouse strains were used: *R26CAGBmp2*^*tg*^ (this report), *Nkx2*.*5*^*Cre*^^12^, *cTnT*^*Cre*^^35^, *Tie2*^*Cre*^^36^ and *BMP2*^*flox*^^49^. For simplicity, *R26CAGBmp2*^*tg/*+^ is abbreviated in the text and figures as *Bmp2*^*tg/+*^. Details of genotyping will be provided upon request. Animal studies were approved by the CNIC Animal Experimentation Ethics Committee and by the Community of Madrid (Ref. PROEX 118/15). All animal procedures conformed to EU Directive 2010/63EU and Recommendation 2007/526/EC regarding the protection of animals used for experimental and other scientific purposes, enforced in Spanish law under Real Decreto 1201/2005.

### ES cell culture, in vitro Cre recombination and EB differentiation

For details see supplemental experimental procedures.

### Immunohistochemistry

For details about antibodies and protocols see supplemental experimental procedures.

### Proliferation analysis and quantification on developing hearts

Cell proliferation in the developing heart was evaluated from BrdU incorporation^50^. For details see supplemental experimental procedures.

### AVC and left ventricle explants

E9.5 WT and transgenic AVCs were harvested in sterile PBS. Left ventricles (lv) were carefully dissected, avoiding contamination with AVC tissue. Explants were placed on collagen gels with the endocardium face down^6^. For details see supplemental experimental procedures.

### Explant culture quantification

For details see supplemental experimental procedures.

### Confocal imaging

Confocal images of E9.5 whole-embryos, stained explants and tissue sections were acquired with a Nikon A1R laser scanning confocal microscope and NIS-Element SD Image Software. Images of stained explants were collected as *z*-stacks. Z-projections and lateral sections were assembled using ImageJ. Images were processed in Adobe Photoshop Creative Suit 5.1.

### RNA-Seq

Hearts of E14.5 WT and *Nkx2*.*5*^*CRE/+*^*; Bmp2*^*tg/+*^ embryos (12 per genotype) were isolated on ice-cold PBS and the atria removed. Tissue was homogenised in Trizol (Invitrogen) using a Tissuelyzer (Qiagen). RNA was pooled into three replicates per genotype. For details see supplemental experimental procedures.

### Accession number

Data are deposited in the NCBI GEO database under accession number GSE100810.

## Electronic supplementary material

Suppl. Information

Suppl. Figure S1

Suppl. Figure S2

Suppl. Figure S3

Suppl. Figure S4

Suppl. Figure S5

Suppl. Figure S6

Suppl. Table S1

Suppl. Table S2

Suppl. Table S3

Suppl. Table S4

Suppl. Table S5

Suppl. File 1
