# Supplementary material for: Myocardial Bmp2 gain causes ectopic EMT and promotes cardiomyocyte proliferation and immaturity
Source: Cell Death Dis. 2018 Mar 14;9(3):399. doi: 10.1038/s41419-018-0442-z (PMC5852166; doi:10.1038/s41419-018-0442-z)
Supplement: Supplementary file 1 — Suppl. Information [file 41419_2018_442_MOESM1_ESM.docx]

**SUPPLEMENTAL INFORMATION**

**Myocardial Bmp2 gain causes ectopic EMT and promotes cardiomyocyte proliferation and immaturity**

Belén Prados, Paula Gómez-Apiñániz, Tania Papoutsi, Guillermo Luxán, Stephane Zaffran, José María Pérez-Pomares & José Luis de la Pompa

^--------------------------------------------------------------------------------------------------------------------------------------------------------------------------------------^**SUPPLEMENTAL MATERIALS AND METHODS**

**Generation of *R26CAGBmp2* transgenic line**

Complete cDNA from mouse *Bmp2* was obtained from clone IMAGE 30846365. The sequence was PCR amplified with Phusion High-Fidelity DNA Polymerase (NEB) and primers containing *BamH*I and *Stu*I sites and was cloned into the *BamH*I and *EcoR*V sites of a *pCDNA3.1-IRESeGFP* plamid previously generated by cloning a *Sal*I IRESeGFP fragment into the *Xho*I site of *pCDNA3.1*. We then modified *pCCALL2* (Lobe et al., 1999) by cloning new *Xba*I sites before and after a CAG cassette, which includes a CMV enhancer/*β-actin* promoter and a rabbit β-Globin polyA signal. The PCR *Bmp2-IRESeGFP* fragment was digested with *BamH*I-*Not*I and cloned into the *Bgl*II-*Not*I sites of the modified *pCCALL2*. We obtained the *Xba*I-cassette containing *CAG-loxP-β-Geo-loxP-Bmp2-IRESeGFP* by digestion and cloned into the *Xba*I site of *pROSA26-1* plasmid (Soriano, 1999). The final construct was linearized with *Xho*I and electroporated into G4 mESCs derived from a cross of 129S6/SvEvTac and C57BL/6Ncr mice (Vintersten et al., 2004). After G418 (200 μg/ml) selection for 7 days, 196 clones were picked. Homologous recombination was identified by Southern blot of *EcoR*V-digested DNA and hybridized with 5’ and 3’ probes (Suppl. Figure S1A). About 20% of the clones were positive, and we selected three clones to confirm karyotype. One positive clone was injected into C57/BL6C blastocysts to generate chimaeras that transmitted the transgene to their offspring. The resulting founders were genotyped by PCR of tail genomic DNA samples using primers in the *R26* locus before and after the cloning site and in the polyA signal of the transgene (see Suppl. Fig.1).

**ES cell culture and *in vitro* Cre recombination**

The mouse ESCs used to generate the *R26CAGBmp2* (*Bmp2^tg/+^*) transgenic line were cultured on mytomicin-treated feeder layer of mouse embryonic fibroblast (MEFs) in ES medium containing DMEM-Glutamax (Invitrogen) supplemented with 20% fetal bovine serum (FBS, Linus), 1000 U/ml leukaemia inhibitory factor (LIF), 0.1 mM non-essential aminoacids (NEAA; Invitrogen) and 100 μM β-ME (Sigma). Cells were electroporated wih a *pCMV-CRE* plasmid, and GFP expressing ESC clones were selected as Bmp2-expressing ES cells (Bmp2-ESC). Non-electroporated mESCs were used as control ESCs.

**EB differentiation**

Control or Bmp2-ESCs were cultured for 3 days and dissociated to form EBs by the hanging drop method (Boheler et al., 2005). Hanging drops containing 800 cells were cultured for 3 days in 20 μl differentiation medium (DMEM-Glutamax (Invitrogen) supplemented with 15% fetal bovine serum (FBS, Linus), 1000 U/ml leukaemia inhibitory factor (LIF), 0.1 mM non-essential aminoacids (NEAA; Invitrogen), and 100 μM β-ME (Sigma). EBs were collected and cultured on a non-adherent petri dish for 2 days. EBs were then plated individually on 0.1% gelatin-coated 96-well plates. For Noggin rescue experiments, control and *Bmp2*-ESCs were treated with 0, 150, 250, 500, or 750 ng/ml Noggin (Peprotech) from d-3 to d17 (d0= day of hanging drop formation). At 150 ng/ml, Noggin did not induce detectable changes in Bmp2-expressing EBs, whereas 750 ng/ml Noggin dramatically affected cardiomyocyte differentiation of control EBs (data not shown). At 250 ng/ml, Noggin did not affect control EB size but did reduce both the expression of all markers analysed and beating ability (data not shown). In Bmp2-expressing EBs, 250 ng/ml Noggin significantly reduced EB size at d3 but not at d5, and did not affect marker expression or restore beating ability (data not shown). For treatment with human recombinant BMP2, EBs were cultured in the presence or absence of BMP2 (20ng/ml; R&D) from the day of collection until the end of the experiment (d3-d17). Samples were collected for posterior processing at d0 (ESCs) and d3, d5, d7, d10, d14, and d17 (EBs).

**ELISA**

Medium was collected from control or *Bmp2*-ESCs after 24 h in culture. Bmp2 released from *Bmp2*-ESCs was measured using the human BMP2 ELISA construction kit (Antigenix America Inc.).

**EB diameter quantification**

We used a Nikon SMZ800N scope and NIS-Element SD Image Software to capture at least 3 images of petri dishes containing EBs in suspension at d3 and d5. Mean EB diameter in each condition was calculated measured using Image J software (<https://imagej.nih.gov/ij/> ) from 10 EBs per picture. Statistical comparisons were made by one-way ANOVA test, followed by a Bonferroni post-test. Data are presented as means ± SD. Differences were considered statistically significant at *P* <0.05.

**Proliferation analysis and quantification on EB sections**

EBs at d3 or d5 were either fixed in 4% paraformaldehyde (PFA) directly or after treatment in suspension for 30 min with BrdU (100 μg/ml). Fixed EBs were paraffin embedded and sectioned, and sections were stained with anti-BrdU monoclonal antibody (1:100; Sigma, Ref: B2531) or anti-phospho-histone H3, PH3 (1:100; Upstate; 06-570). Signal was detected with DAB. Proliferation index was calculated as the ratio of PH3- or BrdU-positive nuclei to total cell number counted on non-consecutive sections (≥ 3) of 4 EBs per genotype using Image J. Statistical analyses were performed using unpaired two-tailed t-test. Data are presented as means ± SD. Differences were considered statistically significant at *P* <0.05.

**Quantitative RT-PCR analysis of EBs**

RNA from EBs was purified by Direct-Zol RNA miniprep (Zymo). RNA was pooled to form 3 replicates (d3, 90 EBs; d5, 60 EBs; d7, 12 wells of 96-well plated EBs; d10 and d14, 8 wells of 96-well plated EBs). First-strand cDNA were synthesized with the High Capacity cDNA Reverse Transcription Kit (Applied Biosystems) using 0.5 μg total RNA per reaction. qPCR was performed with Power SYBR Green Master Mix (Applied Biosystems, 4367659). Primers were purchased from KiCqStartTM Primers (Sigma) and are detailed on Supplemental Table 5. A minimum of 2 experiments were performed, with triplicate samples in each experiment.

**Scanning electron microscopy**

Mouse E14.5 hearts were dissected in PBS, fixed in 4% PFA, 1% glutaraldehyde in PBS, and postfixed in 1% OsO_4_ for 90 min at 4ºC. Hearts were then dehydrated and embedded in Durcupan epoxy resin (Sigma). Ultrathin sections (60 μm) were obtained in a Leica microtome. Sections were contrasted with 2% uranyl acetate and Reynolds lead. Ultrastructure images were acquired with a Gatan Orius200 SC digital camera fitted to a Jeol Jem 1010 Transmission Electronic Microscope.

**Histology and *in situ* hybridization**

Hematoxylin/eosin (H&E) staining was performed according to standard protocols. *In situ* hybridization (ISH) and whole-mount *in situ* hybridization (WISH) were performed as described (de la Pompa et al., 1997; Kanzler et al., 1998). Details of probes will be provided on request.

**Immunohistochemistry**

Paraffin-embedded 7 μm sections of embryonic hearts were citrate-unmasked and stained with the following primary antibodies: P-Smad 1/5 (1:100; 9516, Cell Signaling Technology), MEF20 (1:100, DHFB), Periostin (1:100, Novus Biologicals), Snail1 hybridoma supernatant (1:100, (Franci et al., 2006)), Sox9 (1:100, Santa Cruz Biotechnology, sc-20095), anti-alpha smooth muscle actin (sma)-cy3 (1:200, Sigma Aldrich) and IsolectinB4-AlexaFluor-647 (1:200, ThermoFisher Scientific). Secondary antibodies were as follows: anti-rabbit HRP-coupled secondary antibody (P0448, DakoCytomation; 1:100) or anti-mouse HRP-coupled secondary antibody (P0447, DakoCytomation; 1:100). The signal was tyramide signal amplified (TSA) with coupling to Cy3 (NEL744, Perkin Elmer; 1:100) or fluorescein ((NEL744 or NEL741 Perkin Elmer; 1:100). MF20 was detected with goat-anti-mouse-AlexaFluor488 (1:200, ThermoFisher Scientific). Stained tissue sections were analysed with an Olympus BX51 microscope linked to a Nikon DP71 camera and CellA controller software. Images were processed in Adobe Photoshop Creative Suit 5.1

**pSmad staining quantification**

Staining for pSmad 1/5, MF 20, and isolectinB4 (see above) was quantified in sections from wild type and mutant E14.5 hearts. Total cells and pSmad-positive cells in the AVC, endocardium, and myocardium were counted on non-consecutive sections (≥ 3) from 3 embryos of each genotype using ImageJ software. The pSmad activation index was calculated as the ratio of pSmad-positive nuclei to the total number of nuclei (DAPI positive).

**Total area and total cell number quantification**

Total area of different heart compartments was quantified from images of wild type and mutant hearts stained with pSmad 1/5, MF 20, and isolectinB4 (see above). The area occupied by AVC, trabecular and compact myocardium were measured on non-consecutive sections (≥ 3) in 3 embryos of each genotype using ImageJ. AVC and the left and right ventricles were analysed separately. For each measurement, settings were kept constant. Area measurements were taken in each region and the mean was expressed in μm^2^. The total number of cells (DAPI-positive) in the AVC, endocardium, and myocardium were counted on non-consecutive sections (≥ 3) in the same 3 embryos of each genotype using ImageJ.

**Proliferation analysis and quantification on developing hearts**

Cell proliferation in the developing heart was evaluated from BrdU incorporation (Del Monte et al., 2011). BrdU was detected by staining with a mouse anti-BrdU monoclonal antibody (see proliferation analysis on EBs sections above) followed by anti-mouse-biotinilated secondary antibody (Biotin-SP-AffiniPure F(ab')2 Fragment Goat Anti-Mouse IgG, Jackson ImmunoResearch). The signal was tyramide signal amplified (TSA) with coupling to Cy3 (see above). Sections were then stained with MF 20, and isolectinB4 (see above). Total cells (DAPI) and BrdU-positive cells in the myocardium, in the mesenchyme or in the endocardium of developing hearts were counted on non-consecutive sections (≥ 3) in 3 embryos of each genotype using Image J. Statistical analyses were performed using unpaired two-tailed *t*-test. Data are presented as means ± SD. Differences were considered statistically significant at *P* <0.05.

**AVC and left ventricle explants**

Explants were allowed to attach for o/n at 37ºC, 5%CO_2_ and maintained in culture for 3 days (AVC) or 4 days (lv). All explants were fixed and stained with phalloidin-FITC (1:100, Sigma) to reveal the cytoskeleton, with an antibody to α-smooth muscle actin (α-SMA-Cy3; 1:100, Sigma) to detect mesenchymal cells and anti-Cdh5 to detect endothelial cells (1:100, BD Pharmingen). Explants were then mounted on excavated slides with Vectashield medium containing DAPI.

**Explant culture quantification**

The transformation index of the AVC and ventricular explants was calculated as the ratio of the number of cells scattering (2D migration) or invading the collagen gel (3D migration) to the total number of cells (2D migrating cells + 3D migrating cells + endocardial cells)

2D Transformation Index = 2D / (2D + 3D + endocardial cells)

3D Transformation Index = 3D / (2D + 3D + endocardial cells)

Cell number was determined by counting nuclei in the DAPI z-stack, distinguishing between 2D migrating cells, 3D migrating cells, and endocardial cells. At least 8 explants were assayed per genotype and condition. Data are presented as means + SD. Statistical comparisons were made by unpaired two-tailed t-test to assess differences between two groups. Differences were considered statistically significant at *P*<0.05.

**Quantification of coronary vessels**

An estimation of the number of intramyocardial coronary vessels per ventricle was obtained from the quantification of the number of *Fabp4*-expressing vessels in E14.5 wild type and transgenic hearts, counted in non-consecutive sections (≥ 3) in 4 embryos of each genotype, using ImageJ. Statistical analyses were performed using unpaired two-tailed *t*-test. Data are presented as means ± SD. Differences were considered statistically significant at *P* <0.05.

**RNA-Seq**

RNA was prepared using the standard Illumina TrueSeq RNASeq library preparation kit. Libraries were sequenced in a GAIIx Illumina sequencer using a 75bp single end elongation protocol. Sequenced reads were QC and pre-processed using cutadapt v1.6 (Martin, 2011) to remove adaptor contaminants. We excluded one WT sample because it did not cluster well with the rest of the WT samples. For details of RNA-seq data analysis see Supplemental File 1. Resulting reads were aligned and gene expression quantified using RSEM v1.2.3 (Li and Dewey, 2011) over mouse reference GRCm38 and Ensembl genebuild 70. Gene differential expression was analysed using the EdgeR R package (Robinson et al., 2010). Genes showing altered expression with adjusted *P* value <0.05 were considered to be differentially expressed. For the set of differentially expressed genes, a functional analysis was performed using Ingenuity Pathway Analysis Software and DAVID (Huang da et al., 2009), and some of the enriched processes were selected according to relevant criteria related to the biological process studied. Unsupervised hierarchical clustering with Genesis Software (Sturn et al., 2002) was used to group genes according to the similarity of their expression profiles. Using the new R visualization package GOPlot (Walter et al., 2015), a chord plot (van de Weijer et al., 2012) was generated to better visualize the relationships between genes and the selected enriched processes.

**Quantitative RT-PCR of E9.5 and E14.5 hearts**

First-strand cDNAs were synthesized using the High Capacity cDNA Reverse Transcription Kit (Applied Biosystems); each reaction was conducted with 0.5 μg total RNA pooled into 3 replicates per genotype (see RNAseq). For E9.5, hearts were isolated on ice-cold PBS and pooled into 3 replicates (4 embryos per genotype). Hearts of E14.5 WT and *Tie2^CRE/+;^ Bmp2^tg/+^* embryos (3 per genotype) were isolated on ice-cold PBS. Tissue was homogenized as in RNA-seq section. Quantitative PCR was performed with Power SYBR Green Master Mix (Applied Biosystems, 4367659). Primers are listed in Supplemental Table 5. The rest of the primers used (KiCqStartTM primers) were purchased from Sigma. Experiments were performed with triplicate samples. Statistical analyses were performed using unpaired two-tailed t-test to assess differences between two groups. Data are presented as mean ± SD. A *P* value of <0.05 was considered significant.

**SUPPL. FIGURE LEGENDS**

**Suppl. Figure S1. Gene targeting strategy and morphological characterization of *Nkx2.5^Cre/+^;Bmp2^tg/+^* hearts. A,** *Left*, Gene targeting strategy in mouse G4 ESCs. The CAG cassette and a floxed *β-Geo*-STOP cassette followed by the *Bmp2* cDNA and an IRES-*eGFP* were targeted into the *Rosa26* locus. The blue arrows indicate the position of the PCR primers used for genotyping. *Right*, Positive ESC clones analysed by Southern blot of *EcoR*V-digested genomic DNA using 5’ and 3’ probes that identify a 5.4Kb and a 15.6Kb transgenic bands, respectively. **B**, H&E staining of transverse sections of E9.5, E12.5 and E14.5 WT and *Nkx2.5^Cre/+^;Bmp2^tg/+^* hearts, showing general views and details of the atrio-ventricular canal (avc) and left ventricle (lv) at E9.5, the right ventricle (rv) and interventricular septum (ivs) at E12.5, and the right ventricle (rv) and aortic valve (av) at E14.5. Arrowheads point to mesenchymal cells in the avc or av of the E9.5 and E14.5 WT heart, respectively, to the ectopic mesenchymal cells in the rv of the E9.5 *Nkx2.5^Cre/+^;Bmp2^tg/+^* heart, and to the enlarged av region of the E14.5 *Nkx2.5^Cre/+^;Bmp2^tg/+^* heart. Arrows indicate the normal trabeculae in the E12.5 and E14.5 WT hearts and the dysmorphic trabeculae in *Nkx2.5^Cre/+^;Bmp2^tg/+^* hearts. Scale bar, 200 μm. **C**, transmission electron microscopy (TEM) images of E14.5 WT ventricular trabeculae, showing loosely organized cardiomyocytes (arrowheads) overlaid by flattened endocardial cells (arrows). **D**, TEM images of E14.5 *Nkx2.5^Cre/+^;Bmp2^tg/+^* trabeculae packed with cardiomyocytes (arrowheads) and overlaid by rounded endocardial cells (arrows). Detailed views show the well-organized Z band structure in the WT sarcomere (**C’-C’’**, white arrowheads) and the less organized structure in the *Nkx2.5^Cre/+^;Bmp2^tg/+^* sarcomere (**D’-D’’**, white arrowheads).

**Suppl. Figure S2. Myocardial *Bmp2* expression rescues the AVC specification defect in *Bmp2*-deficient mice. A**, qRT-PCR analysis in E14.5 WT and *Nkx2.5^Cre/+^;Bmp2^tg/+^* hearts. **B,** *Top*, Whole-mount view of the heart in control (*Bmp2^flox/flox^*), *Nkx2.5^Cre/+^;Bmp2^flox/flox^*, *Nkx2.5^Cre/+^;Bmp2^tg/+^*;*Bmp2^flox/flox^*, and *Nkx2.5^Cre/+^;Bmp2^tg/+^* E9.5 embryos. The brackets demarcate the AVC canal that is missing in the *Nkx2.5^Cre/+^;Bmp2^flox/flox^* mutants (arrowhead). *Middle*, H&E staining of transverse sections as in **A**. Black and white arrowheads point to mesenchyme cells in the AVC and ventricles, respectively. *Bottom*, ISH showing *Bmp2* expression in the AVC of the various genotypes (arrowheads). **C**, H&E staining and *Bmp2* ISH in control (*Bmp2^flox/flox^*) and *Nkx2.5^Cre/+^;Bmp2^tg/+^*;*Bmp2^flox/flox^* E14.5 hearts. Scale bars, 200 μm. Abbreviations as in Suppl. Figure S1; tv, tricuspid valve; mv, mitral valve. **D**, qRT-PCR analysis of EMT drivers and patterning genes in E9.5 WT and *Nkx2.5^Cre/+^;Bmp2^tg/+^* hearts.

**Suppl. Figure S3. Regionalized expression of EMT drivers and ECM components and increased proliferation in E14.5 *Nkx2.5^Cre/+^;Bmp2^tg/+^* hearts. A,** Snail, *Slug*, *Twist1*, Sox9, *Has2*, *Tgfβ2* and periostin expression demarcates the expanded valve territory in *Nkx2.5^Cre/+^;Bmp2^tg/+^* hearts. Snail, Sox9 (red) and periostin (green) are immunostainings, the rest are ISH. Nuclei were counterstained with DAPI (blue) in immunostainings. Arrowheads point to the valve mesenchyme. Abbreviations as in Suppl. Figure S1. mv, mitral valve; tv, tricuspid valve. **B**, BrdU incorporation analysis in E14.5 WT and transgenic heart sections. Arrowheads and arrows mark BrdU-positive (red) mesenchyme and myocardial nuclei, respectively. Nuclei are counterstained with DAPI (blue). AV, aortic valve; LV, left ventricle. Scale bars, 200 μm. **C**, Proliferation index in E12.5 WT and transgenic hearts (in which the various cell types have been specifically labelled, see Figure 4B) and E14.5 WT and transgenic hearts.

**Suppl. Figure S4. Reduced coronary vessels markers expression in *Nkx2.5^Cre/+^;Bmp2^tg/+^*** **embryos.** ISH of the coronary artery markers *Dll4* (**A,B**), *Hey1* (**C,D**), and *Cxcr4* (**E,F**) and of the pan-coronary marker *Fabp4* (**G,H**). Arrowheads point to developing coronary vessels. Scale bars, 200 μm. **I**, quantification of the number of *Fabp4*-positive coronary vessels in WT and transgenic embryos.

**Suppl. Figure S5. Ectopic Bmp2 expression in myocardium, but not endocardium, disrupts cardiogenesis. A**, *left*, Parasagittal sections of H&E-stained E10.5 WT and *cTnT^Cre/+^;Bmp2^tg/+^* hearts. Detailed views of the AVC are shown in the lower panels. Black arrowheads point to mesenchyme cells in the AVC. White arrowheads point to ectopic mesenchyme cells beneath the endocardium of the left ventricle. *Right*, Transverse sections of H&E-stained hearts of the same genotypes. Detailed views of the AVC and RV are shown in the lower panels. Note the presence of ectopic mesenchyme cells in the right ventricle (white arrowheads). **B-D**, Transverse sections of E14.5 WT and *cTnT^Cre/+^;Bmp2^tg/+^* hearts. **B**, *Left*, H&E staining. Severe dysmorphogenesis in the *cTnT^Cre/+^;Bmp2^tg/+^* heart includes a ventricular septum defect (asterisk), thin compact myocardium, and persistent trabeculae. *Right*, *Bmp2* ISH, showing expression in the AVC region of both genotypes (arrowheads) and more widespread expression in the transgenic heart (arrows). **C**, *Left*, *Tbx20* ISH, showing markedly increased *Tbx20* transcription in ventricles and atria. *Right*, *Hey2* ISH, showing expression in WT compact myocardium (arrow) and valve endocardium (arrowheads); *cTnT^Cre/+^;Bmp2^tg/+^* hearts show expansion to trabeculae (arrows) and relatively normal expression in valves (arrowheads). **D**, *Top left*, *Cx40* is expressed in WT developing coronaries (arrowheads) and trabeculae (arrow) and is markedly reduced in *cTnT^Cre/+^;Bmp2^tg/+^* hearts. *Top right*, *Bmp10* expression in WT trabecular myocardium (arrows) is attenuated in transgenic embryos, especially in the right ventricle. *Bottom left*, *Tbx2* is expressed in the AVC region (arrowheads), which is enlarged in the *cTnT^Cre/+^;Bmp2^tg/+^* heart (arrowheads). *Bottom right*, *Cx43* transcription is unaffected in transgenic embryos. **E**, *Left*, H&E stained parasagittal sections of E10.5 WT and *Tie2^Cre/+^;Bmp2^tg/+^* hearts. *Right*, H&E stained transverse sections of E10.5 hearts of the same genotypes. Chambers and valve territories appear normal. Scale bars, 200 μm.

**Suppl. Figure S6. qRT-PCR analysis in E14.5 *Tie2^Cre/+^;Bmp2^tg/+^* hearts and increased proliferation and defective cardiac differentiation of *Bmp2*-expressing EBs. A,** Quantitative gene expression analysis (qRT-PCR) in E14.5 WT and *Tie2^Cre/+^;Bmp2^tg/+^* hearts. **B**, *Top*, *GFP* expression in Bmp2-expressing ESC (Bmp2-ESCs) after *in vitro* Cre-mediated deletion of the floxed *βGeo-STOP* cassette. *Bottom*, PCR from various ESC clones using primers targeting the *CAG* cassette and *Bmp2* sequence (blue arrows), identifying a 280-bp band after deletion of the floxed *βGeo-STOP* cassette. Positive clones express *Bmp2* and *GFP*. **C,** ELISA showing a 3.5-fold increase Bmp2 in *Bmp2*-ESC culture medium vs. control ESCs. **D,** Morphology of control and Bmp2-EBs expressing *GFP* in suspension at d5, or plated at d7 of cardiac differentiation. **E,** BrdU immunostaining of EBs at d3, showing more BrdU-positive cells in the *Bmp2*-EBs. Scale bar, 100μm. The proliferation index was calculated as the ratio of BrdU-positive cells to the total cell number. ***P*<0.01, *t* test. **F,** qRT-PCR of *GFP* and *Bmp2* and cardiac differentiation markers (*α-Mhc*, *β-Mhc*, *Mlc2a*, *Mlc2v*) not expressed in *Bmp2*-EBs, while *Smad6* is markedly increased. Control EBs (red), *Bmp2*-EBs (green). **G**, qRT-PCR of the specification marker *Mesp1*, the late differentiation markers *β-Mhc*, *Mlc2a* and *Mlc2v* in control EBs (C, red) and human recombinant BMP2-treated EBs (+BMP2, green). Scale bars, 200 μm in B and D, and 100 μm in C.

**Supplementary File 1.** Excell file containing the list of dysregulated genes identified by RNA-seq analysis (sheet one, upregulated genes; sheet two, downregulated genes).

**SUPPLEMENTARY TABLES LEGENDS**

**Supplementary Table S1.** Lethality phase of *Nkx2.5^Cre/+^;Bmp2^tg/+^* embryos.

**Supplementary Table S2.** Rescue of *Nkx2.5^Cre^; Bmp2^flox/flox^* lethality by *Bmp2^tg^*.

**Supplementary Table S3.** Lethality phase of *cTnT^Cre/+^;Bmp2^tg/+^* embryos.

**Supplementary Table S4.** *Tie2^Cre/+^;Bmp2^tg/+^* embryos survive to adulthood.

**Supplementary Table S5.** List of primers used for qRT-PCR. The following mouse KiCqStartTM primers were purchased from Sigma: *Brachyury (T), Mesp1, Bmp2, Id1, Id2, Id3, Tbx5, Gata4, Tnn2, Smad6, Has2, Hand1, Hand2, Nppa, Gja1, Tbx2, Twist1, Snai1, Slug, Cdh5, Smad6* and *Tgfβ2.*

**References**

Boheler, K.R., Crider, D.G., Tarasova, Y., and Maltsev, V.A. (2005). Cardiomyocytes derived from embryonic stem cells. Methods Mol Med *108*, 417-435.

de la Pompa, J.L., Wakeham, A., Correia, K.M., Samper, E., Brown, S., Aguilera, R.J., Nakano, T., Honjo, T., Mak, T.W., Rossant, J.*, et al.* (1997). Conservation of the Notch signalling pathway in mammalian neurogenesis. Development *124*, 1139-1148.

Del Monte, G., Casanova, J.C., Guadix, J.A., Macgrogan, D., Burch, J.B., Perez-Pomares, J.M., and de la Pompa, J.L. (2011). Differential Notch Signaling in the Epicardium Is Required for Cardiac Inflow Development and Coronary Vessel Morphogenesis. Circ Res *108*, 824-836.

Franci, C., Takkunen, M., Dave, N., Alameda, F., Gomez, S., Rodriguez, R., Escriva, M., Montserrat-Sentis, B., Baro, T., Garrido, M.*, et al.* (2006). Expression of Snail protein in tumor-stroma interface. Oncogene *25*, 5134-5144.

Huang da, W., Sherman, B.T., and Lempicki, R.A. (2009). Systematic and integrative analysis of large gene lists using DAVID bioinformatics resources. Nat Protoc *4*, 44-57.

Kanzler, B., Kuschert, S.J., Liu, Y.H., and Mallo, M. (1998). Hoxa-2 restricts the chondrogenic domain and inhibits bone formation during development of the branchial area. Development *125*, 2587-2597.

Li, B., and Dewey, C.N. (2011). RSEM: accurate transcript quantification from RNA-Seq data with or without a reference genome. BMC bioinformatics *12*, 323.

Lobe, C.G., Koop, K.E., Kreppner, W., Lomeli, H., Gertsenstein, M., and Nagy, A. (1999). Z/AP, a double reporter for cre-mediated recombination. Dev Biol *208*, 281-292.

Martin, M. (2011). Cutadapt removes adapter sequences from high-throughput sequencing reads. EMBnetjournal *17*, 10-12.

Robinson, M.D., McCarthy, D.J., and Smyth, G.K. (2010). edgeR: a Bioconductor package for differential expression analysis of digital gene expression data. Bioinformatics (Oxford, England) *26*, 139-140.

Soriano, P. (1999). Generalized lacZ expression with the ROSA26 Cre reporter strain. Nature genetics *21*, 70-71.

Sturn, A., Quackenbush, J., and Trajanoski, Z. (2002). Genesis: cluster analysis of microarray data. Bioinformatics (Oxford, England) *18*, 207-208.

van de Weijer, T., van Ewijk, P.A., Zandbergen, H.R., Slenter, J.M., Kessels, A.G., Wildberger, J.E., Hesselink, M.K., Schrauwen, P., Schrauwen-Hinderling, V.B., and Kooi, M.E. (2012). Geometrical models for cardiac MRI in rodents: comparison of quantification of left ventricular volumes and function by various geometrical models with a full-volume MRI data set in rodents. American journal of physiology Heart and circulatory physiology *302*, H709-715.

Vintersten, K., Monetti, C., Gertsenstein, M., Zhang, P., Laszlo, L., Biechele, S., and Nagy, A. (2004). Mouse in red: red fluorescent protein expression in mouse ES cells, embryos, and adult animals. Genesis *40*, 241-246.

Walter, W., Sanchez-Cabo, F., and Ricote, M. (2015). GOplot: an R package for visually combining expression data with functional analysis. Bioinformatics (Oxford, England) *31*, 2912-2914.
