# Supplementary figures and images for: Myocardial Bmp2 gain causes ectopic EMT and promotes cardiomyocyte proliferation and immaturity
Source: Cell Death Dis. 2018 Mar 14;9(3):399. doi: 10.1038/s41419-018-0442-z (PMC5852166; doi:10.1038/s41419-018-0442-z)

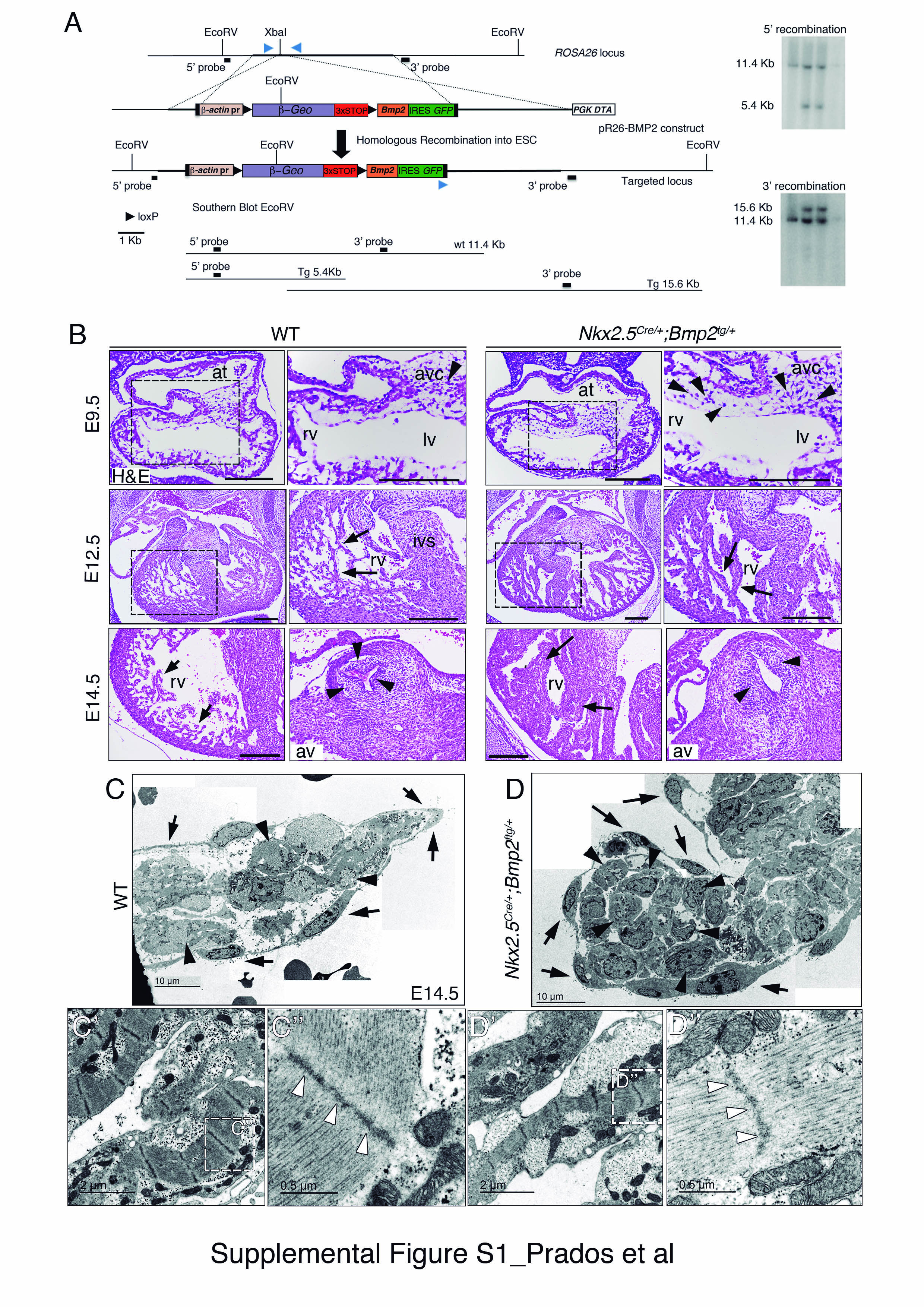

Supplement: Supplementary file 2 — Suppl. Figure S1 [file 41419_2018_442_MOESM2_ESM.jpg]

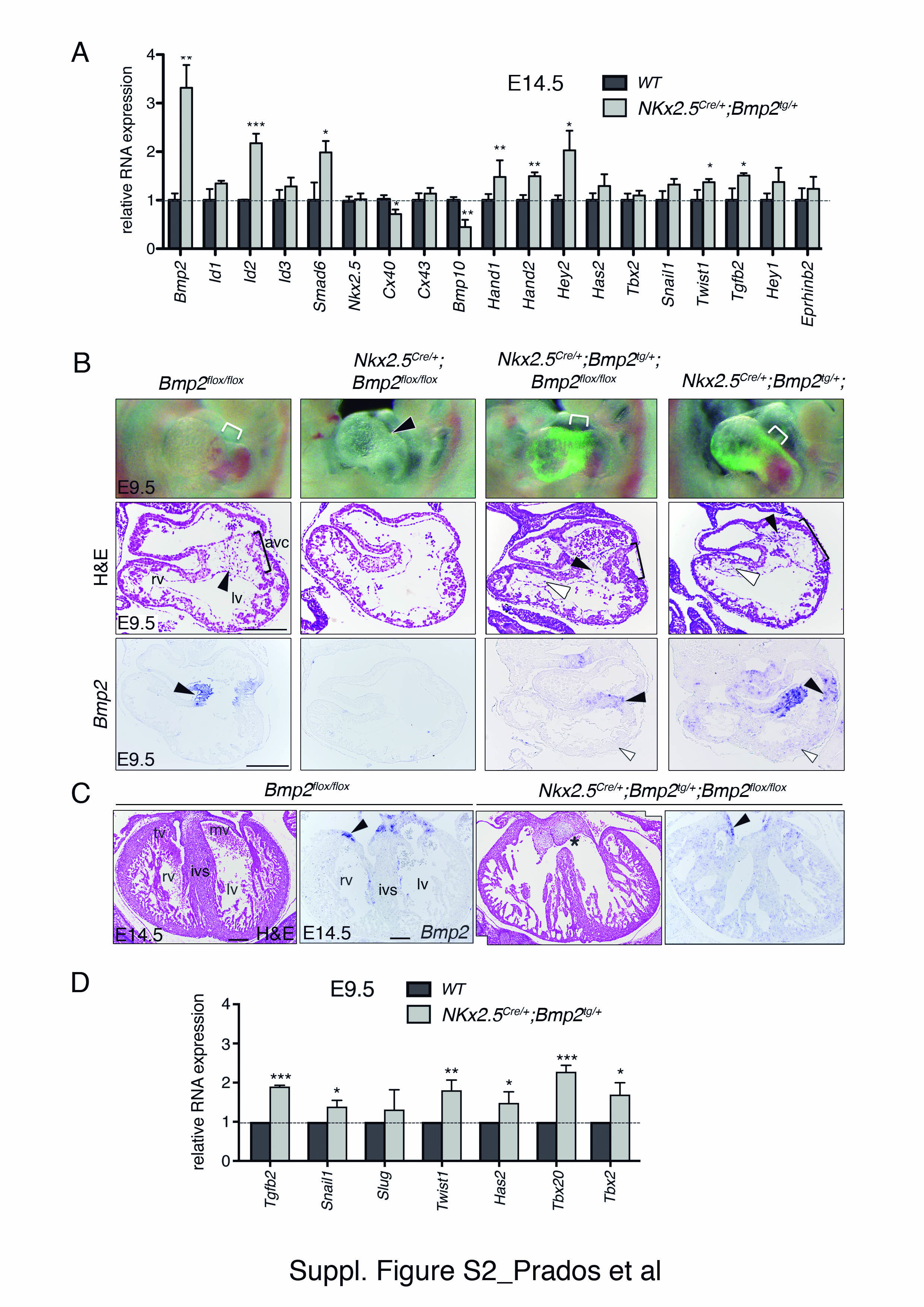

Supplement: Supplementary file 3 — Suppl. Figure S2 [file 41419_2018_442_MOESM3_ESM.jpg]

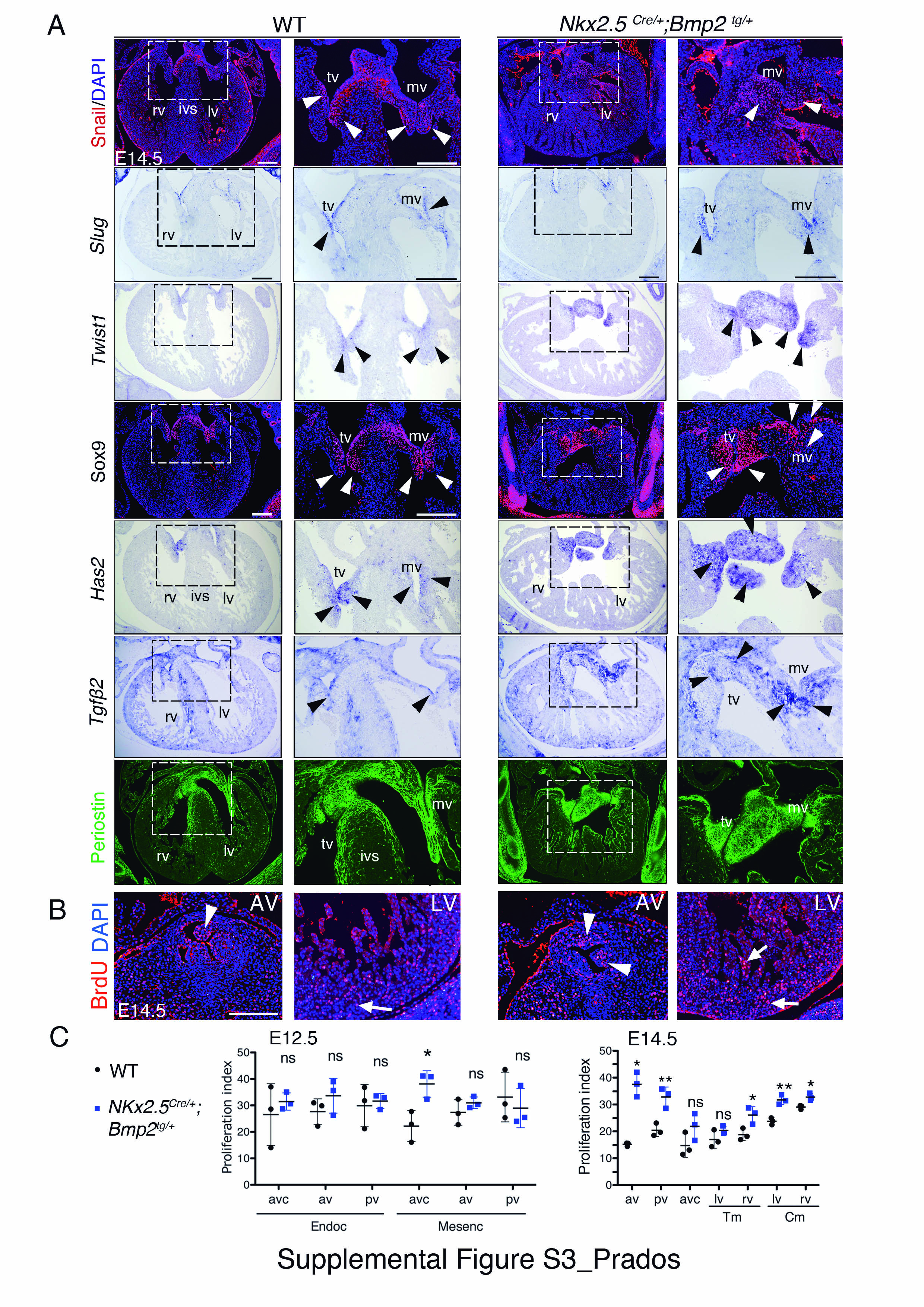

Supplement: Supplementary file 4 — Suppl. Figure S3 [file 41419_2018_442_MOESM4_ESM.jpg]

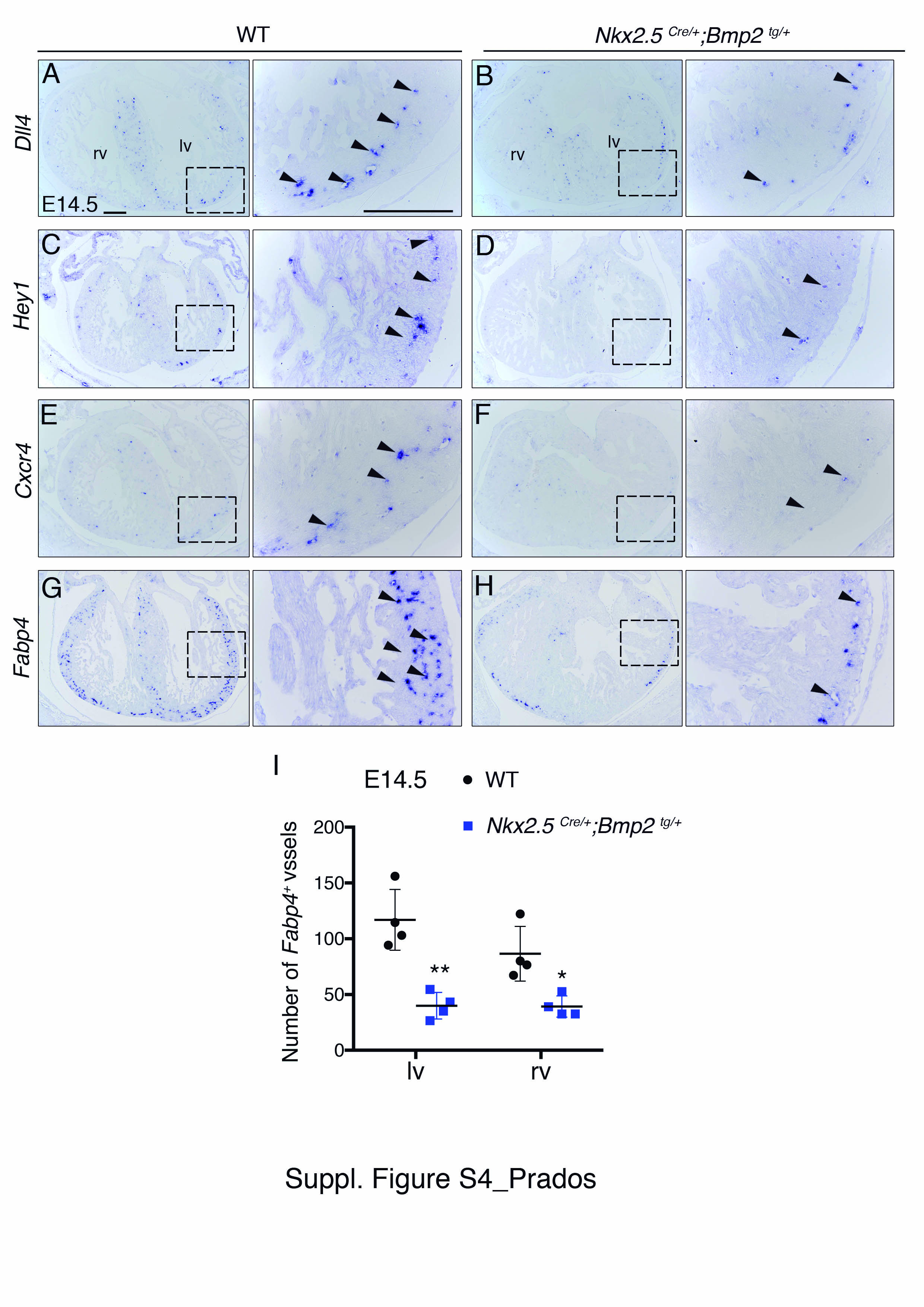

Supplement: Supplementary file 5 — Suppl. Figure S4 [file 41419_2018_442_MOESM5_ESM.jpg]

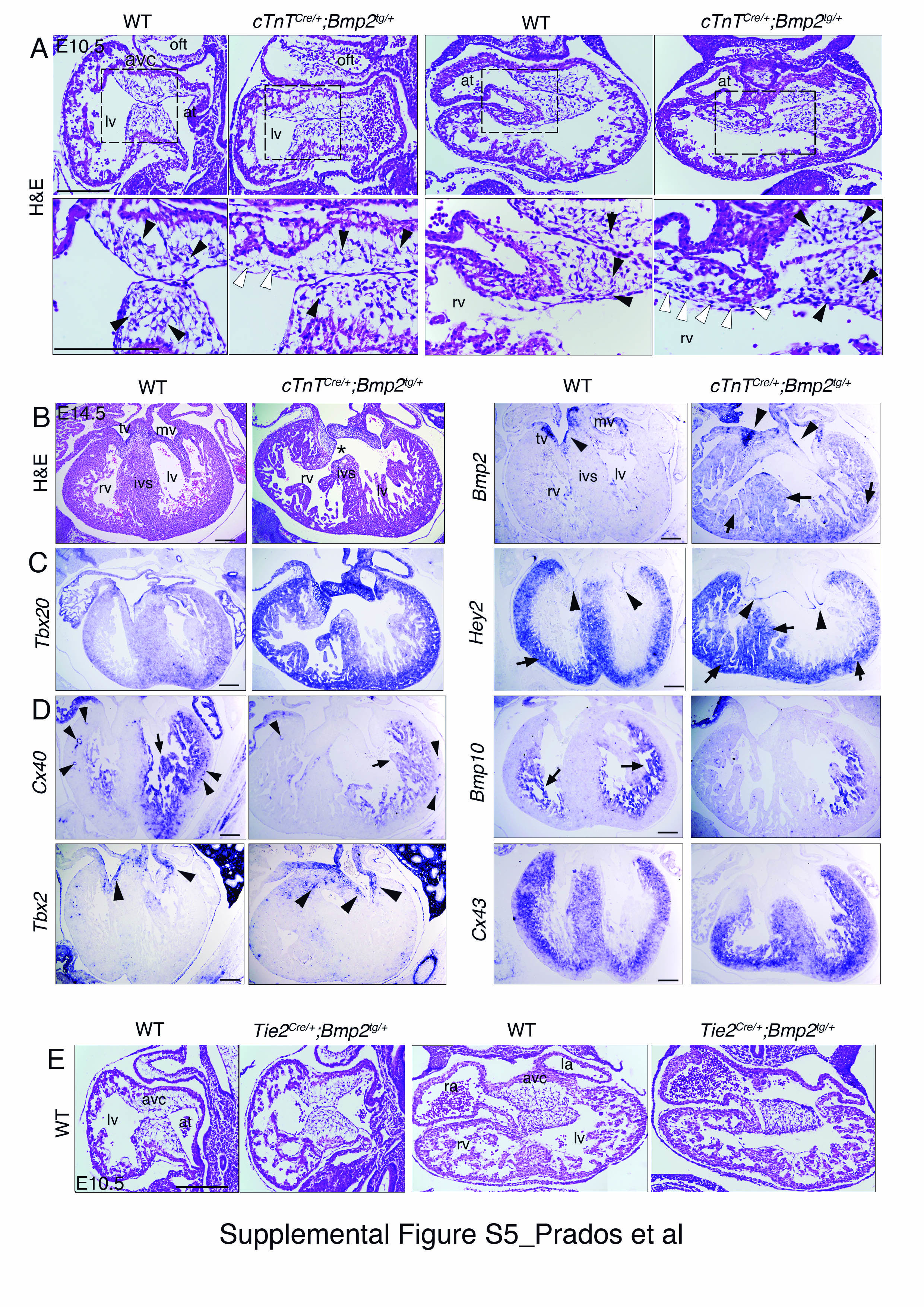

Supplement: Supplementary file 6 — Suppl. Figure S5 [file 41419_2018_442_MOESM6_ESM.jpg]

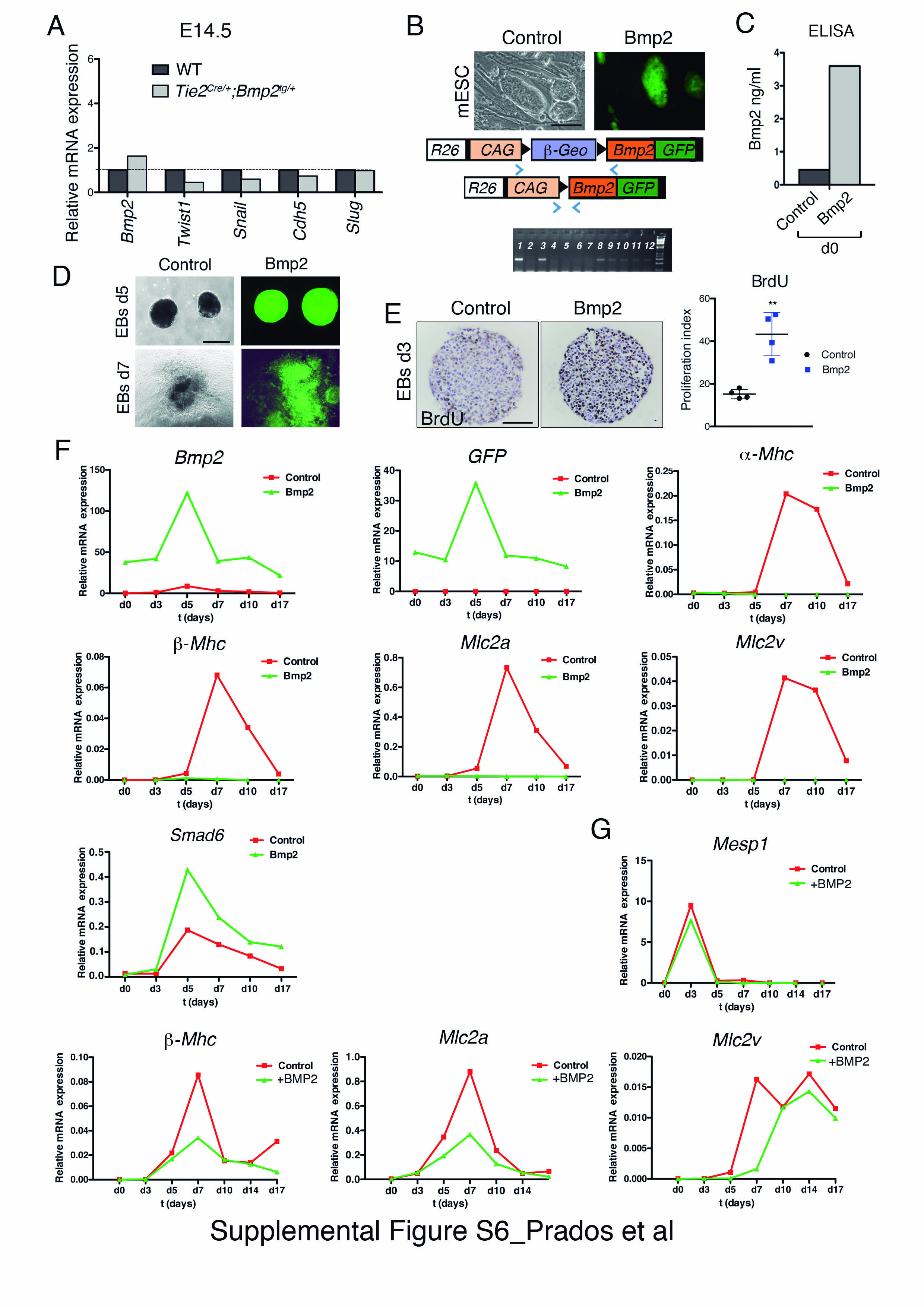

Supplement: Supplementary file 7 — Suppl. Figure S6 [file 41419_2018_442_MOESM7_ESM.jpg]
