# Supplementary material for: Myocardial Bmp2 gain causes ectopic EMT and promotes cardiomyocyte proliferation and immaturity
Source: Cell Death Dis. 2018 Mar 14;9(3):399. doi: 10.1038/s41419-018-0442-z (PMC5852166; doi:10.1038/s41419-018-0442-z)
Supplement: Supplementary file 8 — Suppl. Table S1 [file 41419_2018_442_MOESM8_ESM.docx]

**Supplemental Table S1:** Lethality phase of *Nkx2.5^Cre/+^;Bmp2^tg/+^* embryos. Genotypes obtained after breeding males *Bmp2^tg/+^* with *Nkx2.5^Cre/Cre^* females

|  | Number of embryos | *Nkx2.5^Cre/+^;Bmp2^tg/+^* | *Nkx2.5^Cre/+;^Bmp2^+/+^* |
| --- | --- | --- | --- |
| E9.5 | 135 | 72 (53.3%) | 63 (46.6%) |
| E10.5 | 62 | 33 (53.22%) | 29 (46.77%) |
| E12.5 | 40 | 17 (42.5%) | 23(57.5%) |
| E14.5 | 68 ^*(2,94%)^ | 32 (47.05%) | 34 (50%) |
| E15.5 | 71 ^*12 (16,90%)^ | 16 (22.53%) | 43 (60.56%) |
| E16.5 | 7 ^*3 (42,85%)^ | 0 (0%) | 4 (57.14%) |
| Total | 383 ^*15 (3,91%)^ | 170 (44.38%) | 196 (51.17%) |

* necrotic
