# Supplementary material for: Myocardial Bmp2 gain causes ectopic EMT and promotes cardiomyocyte proliferation and immaturity
Source: Cell Death Dis. 2018 Mar 14;9(3):399. doi: 10.1038/s41419-018-0442-z (PMC5852166; doi:10.1038/s41419-018-0442-z)
Supplement: Supplementary file 9 — Suppl. Table S2 [file 41419_2018_442_MOESM9_ESM.docx]

**Supplemental Table S2:** Rescue of *Nkx2.5^Cre^; Bmp2^flox/flox^* lethality by *Bmp2^tg.^* Genotypes obtained after breeding *Nkx2.5^Cre/Cre^;Bmp2^flox/+^* males with *Bmp2^tg/+^;Bmp2^flox/flox^* females.

|  | Number of embryos  ^*necrotic^  ^#resorptions^ | *Nkx2.5^Cre/+^;*  *Bmp2^+/+^;*  *Bmp2^flox/+^* | *Nkx2.5^Cre/+^;*  *Bmp2^tg/+^;*  *Bmp2^flox/flox^* | *Nkx2.5^Cre/+^;*  *Bmp2^+/+^;*  *Bmp2^flox/flox^* | *Nkx2.5^Cre/+^;*  *Bmp2^tg/+^;*  *Bmp2^+/+^* |
| --- | --- | --- | --- | --- | --- |
| E9.5 | 58^* 2 (3.63%); #3 (5,17%)^ | 9 (15.51%) | 14 (24.13%) | 14 (24.13%) | 16 (27.58%) |
| E11.5 | 41^*12 (29.26%); #8 (19,51%)^ | 12 (29.26%) | 1(2.43%) | 0 (0%) | 8 (19.51%) |
| E12.5 | 16^*4 (25%); #4 (25%)^ | 3 (18.75%) | 3 (18.75%) | 0 (0%) | 2 (12.5%) |
| E14.5 | 38^*6 (15.78%); #8 (21,05%)^ | 8 (21.91%) | 5 (13.15%) | 0 (0%) | 11 (28.94%) |
| total | 153^*24 (15.68%); #23 (25,03%)^ | 32 (20.91%) | 23 (15.03%) | 14 (9.15%) | 37 (24.18%) |
