# Supplementary material for: Myocardial Bmp2 gain causes ectopic EMT and promotes cardiomyocyte proliferation and immaturity
Source: Cell Death Dis. 2018 Mar 14;9(3):399. doi: 10.1038/s41419-018-0442-z (PMC5852166; doi:10.1038/s41419-018-0442-z)
Supplement: Supplementary file 10 — Suppl. Table S3 [file 41419_2018_442_MOESM10_ESM.docx]

**Supplemental Table S3:** Lethality phase of *cTnT^Cre/+^;Bmp2^tg/+^* embryos. Genotypes obtained after breeding males *cTnT^Cre/+^ with* female*s Bmp2^tg/+^ .*

|  | Number of embryos  ^*necrotic^ | *cTnT^Cre/+^;*  *Bmp2^tg/+^* | *cTnT^+/+^;*  *Bmp2^tg/+^* | *cTnT^Cre/+^;*  *Bmp2^+/+^* | *cTnT^+/+^;*  *Bmp2^+/+^* |
| --- | --- | --- | --- | --- | --- |
| E9.5 | 33 | 7 (21.21%) | 7 (21.21%) | 8 (24.24%) | 11 (33.33%) |
| E10.5 | 33 | 11 (33.33%) | 12 (36.36%) | 6 (18.18%) | 4 (12.12%) |
| E14.5 | 43 | 10 (23.25%) | 11 (25.58%) | 9 (20.93%) | 13 (30.23%) |
| E15.5 | 51  ^*4 (7.84%)^ | 13 (25.49%) | 11 (21.56%) | 11 (21.56%) | 12 (23.52%) |
| E16.5 | 65  ^*5 (7.69%)^ | 12 (18.46%) | 16 (24.61%) | 18 (27,69%) | 14 (21,53%) |
| E17.5 | 48  ^*2 (4,16%)^ | 8 (16,66%) | 13 (27%) | 16 (33,33%) | 9 (18,75%) |
| Total | 273  ^*11 (4,02%)^ | 61 (22,34%) | 70 (25,64%) | 68 (24,90%) | 63 (23,07%) |
