# Supplementary material for: Myocardial Bmp2 gain causes ectopic EMT and promotes cardiomyocyte proliferation and immaturity
Source: Cell Death Dis. 2018 Mar 14;9(3):399. doi: 10.1038/s41419-018-0442-z (PMC5852166; doi:10.1038/s41419-018-0442-z)
Supplement: Supplementary file 11 — Suppl. Table S4 [file 41419_2018_442_MOESM11_ESM.docx]

|  | Number of embryos | *Tie2^Cre/+^;Bmp2^tg/+^* | *Tie2^Cre/+^;Bmp2^+/+^* |
| --- | --- | --- | --- |
| E9.5 | 36 | 19 (52.77%) | 17 (47.23%) |
| E10.5 | 30 | 14 (46.67%) | 16 (53.33%) |
| E11.5 | 28 | 13 (46.66%) | 15 (53.34%) |
| E14.5 | 17 | 9 (52.94%) | 8 (47.05%) |
| P21 | 26 | 12 (46.16%) | 14 (53.84) |
| total | 126 | 61 (48.4%) | 65 (51.58%) |

**Supplemental Table S4:** *Tie2^Cre/+^;Bmp2^tg/+^* viability table. Genotypes obtained after breeding *Bmp2^tg/+^* males with *Tie2^Cre/Cre^* females
