# Supplementary material for: Myocardial Bmp2 gain causes ectopic EMT and promotes cardiomyocyte proliferation and immaturity
Source: Cell Death Dis. 2018 Mar 14;9(3):399. doi: 10.1038/s41419-018-0442-z (PMC5852166; doi:10.1038/s41419-018-0442-z)
Supplement: Supplementary file 12 — Suppl. Table S5 [file 41419_2018_442_MOESM12_ESM.docx]

**Supplemental Table S5:** list of primers used for qRT-PCR

| **Name** | **Forward primer 5’-3’** | **Reverse primer 5’-3’** |
| --- | --- | --- |
| *Actin* | ATCTTCCGCCTTAATACT | GCCTTCATACATCAAGTT |
| *Gapdh* | AACTTTGGCATTGTGGAAGG | ACACATTGGGGGTAGGAACA |
| *GFP* | TCGTGACCACCCTGACCTAC | TCTTGTAGTTGCCGTCGTCCT |
| *Gusb* | ACTCCTACTGAACATGCGA | ATAAGACGCATCAGAAGCCG |
| *n-Myc* | GGAAGTTCACACCTAAGT | AGTTATGTATCAGCGTCAT |
| *Hey1* | CTCTGCCTTCTCATTCATT | TAGTCACAACACATCAATACA |
| *Nkx2.5* | GGCTTGTCCAGCTCCACT | CATTTTACCCGGGAGCCTAC |
| *Bmp10* | ACCAAGCTGAGGACACCGGAAGG | CTTCGTGGGCACACAGCAGGCTTT |
| *MyL2* | TGGGTAATGATGTGGACCAA | CTCACACTCTTCGGGGAGAA |
| *Myh6* | CTTCATCCATGGCCAATTCT | GCGCATTGAGTTCAAGAAGA |
| *Myh7* | GAGCCTTGGATTCTCAAACG | GTGGCTCCGAGAAAGGAAG |
| *EphB2* | CGGACTACACCAGCTTTAAC | GCGCATTGAGTTCAAGAAGA |
| *Gja5* | AGCAACATACCAGATAGA | TGTCACTATGGTAGCCCTGAG |
